# The Development and Characterization of a Next-Generation Oncolytic Virus Armed with an Anti-PD-1 sdAb for Osteosarcoma Treatment *In Vitro*

**DOI:** 10.3390/cells13040351

**Published:** 2024-02-17

**Authors:** Theresa A. Higgins, Daniel J. Patton, Isabella M. Shimko-Lofano, Timothy L. Eller, Roberto Molinari, Maninder Sandey, Aliaa Ismail, Bruce F. Smith, Payal Agarwal

**Affiliations:** 1 Scott-Ritchey Research Center, College of Veterinary Medicine, Auburn University, Auburn, AL 36849, USA; tah0076@auburn.edu (T.A.H.); djp0031@auburn.edu (D.J.P.); ims0027@auburn.edu (I.M.S.-L.); tle0018@auburn.edu (T.L.E.); mzs0011@auburn.edu (M.S.); aai0007@auburn.edu (A.I.); smithbf@auburn.edu (B.F.S.); 2 Department of Mathematics and Statistics, College of Sciences and Mathematics, Auburn University, Auburn, AL 36849, USA; robmolinari@auburn.edu; 3 Department of Pathobiology, College of Veterinary Medicine, Auburn University, Auburn, AL 36849, USA; 4 Department of Pathology, Faculty of Veterinary Medicine, Suez Canal University, Ismailia 8366004, Egypt

**Keywords:** conditionally replicative adenovirus (CRAd), cancer immunotherapy, osteosarcoma, immune checkpoint inhibitor, oncolytic adenovirus, PD-1, PD-L1

## Abstract

Osteosarcoma (OS) is a primary bone malignancy characterized by an aggressive nature, limited treatment options, low survival rate, and poor patient prognosis. Conditionally replicative adenoviruses (CRAds) armed with immune checkpoint inhibitors hold great potential for enhanced therapeutic efficacy. The present study aims to investigate the anti-tumor efficacy of CAV2-AU-M2, a CAV2-based CRAd armed with an anti-PD-1 single-domain antibody (sdAb), against OS cell lines *in vitro*. The infection, conditional replication, cytopathic effects, and cytotoxicity of CAV2-AU-M2 were tested in four different OS cell lines in two-dimensional (2D) and three-dimensional (3D) cell cultures. CAV2-AU-M2 showed selective replication in the OS cells and induced efficient tumor cell lysis and death. Moreover, CAV2-AU-M2 produced an anti-PD-1 sdAb that demonstrated effective binding to the PD-1 receptors. This study demonstrated the first CRAd armed with an anti-PD-1 sdAb. This combined approach of two distinct immunotherapies is intended to enhance the anti-tumor immune response in the tumor microenvironment.

## 1. Introduction

Cancer remains a global health challenge, necessitating the constant exploration of innovative therapeutic approaches [1]. Osteosarcoma (OS) predominantly affects children and adolescents [2]. It poses significant challenges due to its aggressive nature, high metastatic potential, and chemotherapy resistance [3]. Over the last 20 years, the survival rates of OS have been unchanged, and there are still no therapies explicitly targeting OS patients [4].

Immunotherapies harness the host’s immune system to recognize and eliminate cancer cells [5]. One promising immunotherapeutic modality is the use of immune checkpoint inhibitors, such as antibodies against PD-1 (programmed cell death protein 1). PD-1 is a protein found on the surface of T cells [6]. It helps regulate the immune system by preventing the T cells from attacking healthy cells when bound by its ligands PD-L1 (programmed death-ligand 1) and PD-L2 (programmed death-ligand 2). However, cancer cells can also overexpress PD-L1 and PD-L2 and suppress the immune response in the tumor microenvironment (TME) by binding to the PD-1 on the T cells [7]. Immune checkpoint inhibitors, such as pembrolizumab and nivolumab (anti-PD-1 mAb), act by blocking the interaction between PD-1 and PD-L1 [8]. These drugs unleash the body’s immune system to target and destroy cancer cells by inhibiting the PD-1 pathway [9]. Anti-PD-1 mAbs have shown significant efficacy in multiple types of cancer, including melanoma, lung, kidney, bladder, and others [10,11,12]. However, the response to anti-PD-1 inhibitors can vary among patients, and they are not effective for all types of cancer. It is important to note that anti-PD-1 inhibitors are typically used in combination with other cancer treatments, such as chemotherapy or targeted therapies, to improve their effectiveness [12].

The role of anti-PD-1 immune checkpoint inhibitors in the treatment of OS is a promising area of active investigation [13]. Arming CRAds with immune checkpoint inhibitors holds great potential for synergistic effects. This integrative/synergistic combinatorial approach using CRAds and immunotherapy offers a multifaceted attack on tumors, targeting both the tumor cells directly and leveraging the immune system for long-lasting anti-tumor effects. This approach will also facilitate the secretion of anti-PD-1 sdAbs locally in the TME and therefore will prevent adverse effects due to the systemic delivery of anti-PD-1 mAbs.

Conditionally replicative adenoviruses (CRAds) are promising cancer therapeutic tools due to their selective replication in tumor cells, efficient gene delivery, robust transgene expression, broad tissue tropism, and ability to induce a potent immune response to tumor cells [14,15]. CRAds have shown potential as a treatment modality for OS in preclinical studies [16]. CRAds lyse tumor cells and induce systemic anti-viral immunity along with anti-tumor immunity. They can promote immunogenic cell death, enhance antigen presentation, and stimulate immune cell infiltration, thereby potentiating the overall therapeutic response [17]. Strategies such as genome modifications to enhance the tumor selectivity, the incorporation of therapeutic transgenes, and combination with other cancer therapies have been explored to maximize the anti-tumor potential of CRAds [18]. These advanced CRAds are used in clinical trials for various cancer types, including melanomas, prostate cancer, glioblastomas, and ovarian cancer [19,20,21,22].

*In vitro* studies have demonstrated the significant oncolytic activity of CRAds against OS [23]. In this context, our laboratory has previously produced two CRAds (CAV2-AU-M1 and CAV2-AU-M2), which were tested for their infectious and transductional advantages in multiple canine cell lines [24]. Herein, we specially verified the oncolytic and cytopathic properties of CAV2-AU-M2 in an OS cell culture. Furthermore, since CAV2-AU-M2 is armed with an anti-PD-1 single domain antibody (sdAb), we demonstrated CAV2-AU-M2′s ability to produce an anti-PD-1 sdAb after infecting the OS cells. This work is a primary step in combining gene therapy and immunotherapy, where CRAds designed to produce anti-PD-1 antibodies directly in the tumor microenvironment enable the T cells to kill the tumor cells and bypass the side effects of the systemic administration of anti-PD-1 sdAbs [25].

## 2. Materials and Methods

### 2.1. Cell Lines and Culture

The canine osteosarcoma cell lines D17, CF11, D22, and MCKOS (a generous gift from Dr. Rimas Orentas, Caring Cross) and the primary normal canine fibroblast (NCF) and human embryonic kidney (HEK 293) cells expressing anti-PD-1 (a generous gift from Dr. Maninder Sandey, Auburn University) were cultured at 37 °C in 5% CO_2_ in Dulbecco’s Modified Eagle’s Medium (DMEM; Corning: cat#10-013-CV, Corning, NY, USA) supplemented with 10% heat-inactivated fetal bovine serum (Thermo, Waltham, MA, USA): cat#16000-044), penicillin (100 IU/mL, Corning: cat#30-002-CI), streptomycin (100 μg/mL, Corning: cat#30-002-CI), and amphotericin B (0.5 µg/mL Corning: cat#30-003-CF). All the cell cultures were tested regularly for mycoplasma contamination using PCR.

### 2.2. Adenoviral Vectors

The genetically modified adenoviruses CAV2-AU-M1 (CAV2-E1A-Δ21-RGD-DsRed) and CAV2-AU-M2 (CAV2-E1AΔ21-RGD-DsRed-ΔE3-anti-PD1) were generated from the pICOCAV15 plasmid as the backbone using CRISPR/Cas9 technology, as published previously [24]. The anti-PD-1 sdAb sequence was inserted under the control of the CMV promoter by substituting E3 in CAV2-AU-M2 [24]. The anti-PD-1 sdAbs were developed by immunizing llamas with canine PD-1 and PD-L1 proteins (QVQuality, Utrecht, The Netherlands). Bacteriophage libraries were produced and selected against canine PD-1 and PD-L1. A diverse pool of sdAbs produced from selected clones were measured for their binding affinity with PD-1 and PD-L1. Selected clones were subcloned to include FLAG and His tags at the C-terminus of the sdAb sequence. We chose the sequence of the sdAb with the highest binding affinity to canine PD-1 (APD-1D5).

### 2.3. Oncolytic Virus Infections

2D cell culture: The canine oncolytic adenovirus CAV2-AU-M2 was used for the infections. All the canine osteosarcoma cell lines and NCF cells were seeded at a density of 2.5 × 10^5^ cells per well in 1 mL of media in a 12-well plate. After 24 h of incubation at 37 °C, the cells were washed with 1× PBS (Phosphate Buffer Saline, Corning: cat#21-040-CV) and infected with the virus at 4 different multiplicities of infection (MOI; 0, 10, 100, 1000) in low-serum DMEM (2% F.B.S.) with no antibiotics. Cell images were taken every 24 h (0, 24, 48 and 72 h) using a Keyence BZ-X800 imaging system.

3D OS spheroid cultures and infections: A total of 5000 canine osteosarcoma or NCF cells were plated in a low-attachment round-bottom 96-well plate (Corning) in 50 µL of complete media along with 50 µL of ECM (30 µg/mL; Collagen I, Thermo: cat#5056). The plates were centrifuged at 500× *g* for 5 min. The cells were incubated at 37 °C and routinely monitored for spheroid formation. At 72 h, the cells were infected with CAV2-AU-M2 (MOI; 0, 100 and 1000). Images were captured on day 0, day 4, and day 7.

### 2.4. LDH Cytotoxicity Assay

The amount of LDH released into the cell culture media after CAV2-AU-M2 infection was assayed using the LDH-Glo™ Cytotoxicity Assay (Promega, Madison, WI, USA): cat#J2380) using the manufacturer’s instructions. Media samples were collected from the 2D cell culture after infection at 0, 24, 48, and 72 h time points and on days 0, 4, and 7 from the 3D cultures. The cell culture media samples were stored in storage buffer (as per the LDH-Glo™ Cytotoxicity Assay manufacturer’s instructions) at −20 °C. An equal amount (25 µL) of the stored sample, diluted 1:100 in storage buffer, and LDH Detection Reagent was incubated in an opaque 96-well plate for 60 min at room temperature. After incubation, the luminescence of each sample was recorded using a microplate reader (BioTek (Winooski, VT, USA) Synergy H1). Negative control wells (triplicate wells containing media only without cells) were added to determine the background luminescence from the LDH in the media.

### 2.5. CAV2-AU-M2 Infections, Nickel Column Purifications, and Western Blotting

The D17, CF11, D22, and MCKOS cell cultures were grown up to 70–80% confluence. The cells were washed once with 1× PBS. CAV2-AU-M1 and CAV2-AU-M2 were used for infections at 100 MOI in low-serum DMEM (5% F.B.S.). The cells were monitored for red fluorescence using the Keyence BZ-X800 imaging system and harvested after 48 h. The cell culture media and cells were collected separately. The culture media was concentrated using a Protein Concentrator PES, 3k MWCO (Thermo, Waltham, MA, USA): cat#88512) and stored at −80 °C. The collected cells were washed once in 1× PBS and lysed using Pierce RIPA buffer with a 1% halt protease inhibitor cocktail (100×; Thermo: cat#87786) and incubation at −80 °C for 30 min. Th cell lysates were centrifuged at 14,000× *g* at 4 °C after lysis. The supernatant collected after centrifugation was stored at −80 °C. The His-tagged anti-PD-1 sdAb was purified from both the cell lysate and concentrated media samples using the Ni-NTA Spin Kit (QIAGEN, Hilden, Germany): cat#31314) as per the manufacturer’s instructions. The purified His-tagged anti-PD-1 sdAbs were stored at −20 °C.

The purified His-tagged anti-PD-1 sdAbs protein samples were boiled in Lane Marker Reducing Buffer (Thermo: cat#39000) for ten minutes before loading them onto NuPAGE 4–12% Bis-Tris Gel (Invitrogen, Waltham, MA, USA): cat#NP0321) along with Chameleon Duo Pre-Stained Protein Ladder (LI-COR, Lincoln, NE, USA): cat#928-60000). Electrophoresis was performed using 1× NuPAGE MOPS SDS Running Buffer (Thermo: cat#NP0001) for approximately 45 min (210 V) and transferred to a PVDF Membrane (0.45 um; Immobilon-P) using 1× Bolt Transfer Buffer (Thermo: BT00061) for 1 h (20 V). The membrane was blocked for 2 h at room temperature (RT) using block buffer (1× PBS, 0.1% Tween, Fisher Scientific: cat#BP337-500, 5% milk protein, Kroger instant non-fat dry milk). The blot was incubated with the primary antibody, mouse anti-6x-His (1:2000; Thermo: cat# MA1-21315), overnight at 4 °C. The next day, the membrane was washed 3x using wash buffer (1× PBS, 0.1% Tween) for 20 min each. The membrane was incubated for 1 hr at RT with the secondary antibody, IRDye 800 CW goat anti-mouse (1:10000; LI-COR: cat#926-32210), and washed 3×, again, for 20 min each. The membrane was scanned using the LI-COR Odyssey system.

### 2.6. Flow Cytometry

#### 2.6.1. PD-1 Binding Assay

A HEK 293 recombinant cell line expressing PD-1 receptors was used. Flow cytometry was carried out to confirm the PD-1 receptor expression. The cells were maintained in DMEM with 10% heat-inactivated fetal bovine serum. The cells were harvested using trypsin (Corning: cat#25-054-CI) and incubated in 10% blocking buffer (1× PBS + normal mouse serum, ImmunoReagents (Raleigh, NC, USA): cat#SP-002-VX5) for 1 h at RT. After blocking, the cells were incubated with the His-tagged anti-PD-1 sdAbs isolated from all the osteosarcoma cell lines’ lysates and media for 1 h. The cells were incubated with the secondary antibody (Mouse Anti-His Tag APC-conjugated Monoclonal Antibody, R&D: cat#IC050A) for 1 h. Uninfected HEK 293 cells were used as the negative control. The cells were washed 3 times with FACS buffer (1X PBS + 0.1% B.S.A.; bovine serum albumin, VWR: cat#0332, + EDTA, Thermo: cat#1861275) and analyzed for R660-APC-A expression in terms of their flow cytometry (CytoFLEX LX, Beckman Coulter; LSR-II; B.D. Biosciences). The data were analyzed using the FlowJo software version 10.7.1. Three independent PD-1 binding experiments were performed.

#### 2.6.2. PD-1/PD-L1 Inhibition Assay

To assess the inhibition of PD-L1 binding to PD-1, adherent HEK 293 cells expressing PD-1 were detached using trypsin and blocked for 1 h using 10% blocking buffer (normal mouse serum) at RT. The cells were incubated with the anti-PD-1 antibodies purified from the cell lysates and cell media of the CAV-AUM2-infected OS cells for 1 h at RT to allow the antibodies to bind to their respective targets. Subsequently, the cells were washed twice with FACS buffer to remove any unbound antibodies and resuspended with the Zenon AF647-labeled canine PD-L1 protein conjugated with human Fc (Thermo: cat#P21462) for 1 h at RT. After washing them with FACS buffer (1XPBS + 0.1% B.S.A.; bovine serum albumin + EDTA) three times, the cells were analyzed for AF647 expression using flow cytometry (CytoFLEX LX, Beckman Coulter; LSR-II, B.D. Biosciences). The data were analyzed using the FlowJo software version 10.7.1 (BD Biosciences, Franklin Lakes, NJ, USA).

### 2.7. Statistical Analysis

#### 2.7.1. Spheroid Size and Luminance

Using R (Version 4.3.1, R Foundation, Vienna, Austria), for each cell line, we ran a linear regression model to test how the spheroid size and luminance changed over time with respect to the levels of MOI (the details of the model can be found in the Supplemental Data). To control for multiple testing errors, we used a significance level α = 0.05 adapted with Bonferroni correction based on the number of cell lines (i.e., α_b1_ = 0.05/5).

#### 2.7.2. Binding and Inhibition Percentages

When testing the PD-1 binding and PD-L1 inhibition percentages using R (version 4.3.1), for all the cell lines, we used a proportion test to assess whether the percentage was greater under CAV2-AU-M2 compared to NI (non-infected cells) and CAV2-AU-M1, respectively, or lower when compared to the secondary Ab PD-L1 for the inhibition condition. For each condition (i.e., binding media, binding CLs, and inhibition), we compared the *p*-values to a Bonferroni-corrected significance level (i.e., α_b2_ = 0.05/8) since we performed 8 comparisons for each condition.

## 3. Results

### 3.1. Infection, Conditional Replication, and Cytopathic Effects (CPE) of CAV2-AU-M2 in Osteosarcoma Cell Lines in 2D and 3D Cell Cultures

#### 3.1.1. CAV2-AU-M2 Infections in 2D Cell Cultures

**Figure 1:**
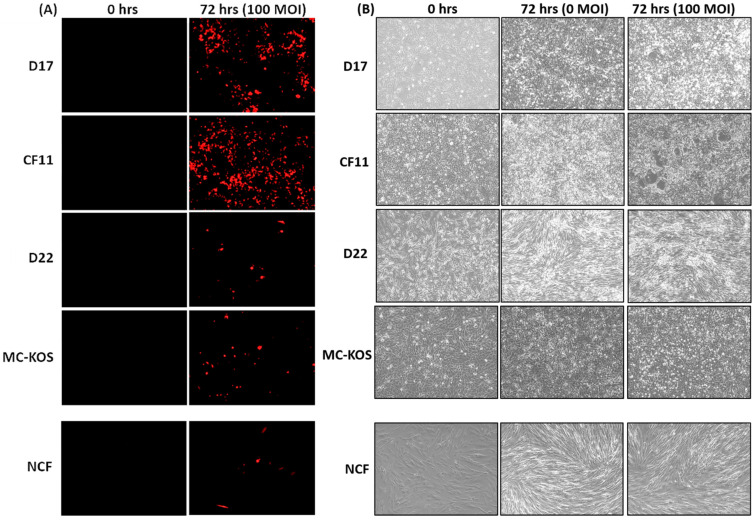
CAV2-AU-M2’s infectious and cytolytic properties in canine osteosarcoma cells in 2D culture. Canine OS cell lines (D17, CF11, D22, and MC-KOS) and NCF cells were infected with CAV-AU-M2 at MOI of 100, and the DsRed fluorescent signal and cytopathic effects in cells were visualized using inverted fluorescent microscopy (Keyence) at 72 h post-infection at 10× magnification. (**A**) DsRed expression. (**B**) Cytopathic effect in infected cells. This is a representation of three independent experiments.

CAV2-AU-M2 is a modified canine adenovirus with E3 deleted and an anti-PD-1 sdAb and DsRed encoding sequences inserted. The purpose of this experiment was to evaluate whether modified CAV2-AU-M2 retained the same infection efficiency, conditional replication, and oncolytic activity in canine osteosarcoma (OS) cell lines as its precursor, ICOCAV15. The oncolytic activity of CAV2-AU-M2 was demonstrated in a wide variety of canine OS cell lines (D17, CF11, D22, and MC-KOS) to represent interindividual tumor heterogeneity. The OS cells, in addition to primary NCF (Normal Canine Fibroblast) cells as a non-cancerous primary cell control, were cultured in 2D and infected with CAV2-AU-M2 at three increasing multiplicities of infection (MOI; 0, 10, 100, and 1000). The infection efficiency of CAV2-AU-M2 was determined by visualizing the red fluorescence every 24 h post-infection using a Keyence BZ-X800 imaging system (Supplementary Data). Red fluorescence indicates virus infection in the cells. Infection with CAV2-AU-M2 (100 MOI) resulted in an increased red fluorescence by 72 h in the OS cells compared to the non-infected cells (0 MOI) and NCF cells (Figure 1A, Supplementary Figures S1–S5). Increased red fluorescence over time in the OS cells in comparison to the NCFs indicated conditional replication and the release of CAV2-AU-M2 for further infection into the neighboring cells. The same effect was absent in the NCFs (Figure 1 A,B). It was also noted that red fluorescence was more evident in D17 and CF11 at 72 h in comparison to the D22 and MC-KOS cells. This may be due to the tumor heterogeneity between different cell lines.

The CAV2-AU-M2-infected OS cell lines D17 and CF11 exhibited a noticeable decrease in cell confluence, which was evident due to the increased space between cells, and increased cell death was evidenced by cell shrinkage and cell rounding at MOI of 100 by 72 h (Figure 1B, Supplementary Figures S6–S10. At MOI of 1000, the infected cells appeared rounded and detached from the bottom of the culture dish, notably representing the typical CPE induced by CAV2-AU-M2 (Supplemental Figures S6–S10). There were no CPE noted in the MC-KOS and D22 cells at MOI of 100 or 1000 (Figure 1B and Supplementary Figures S6–S10). The MC-KOS cells did show some signs of stress post-infection but no visible cell lysis. The NCF cells exhibited cell growth, cell confluence, and a characteristic spindle shape with round to oval nuclei throughout the duration of the experiment and did not show active CPE, suggesting that CAV2-AU-M2 had no lytic effects in the non-cancerous cells (Figure 1B).

**Figure 2:**
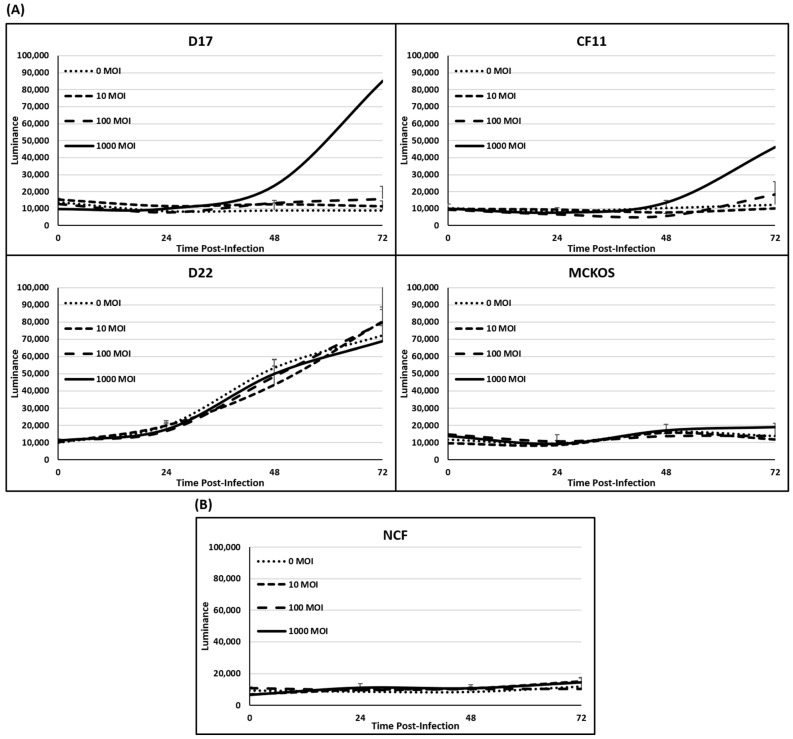
LDH release assay in 2D culture. (**A**) LDH release was measured in cells infected with CAV-AUM2 at MOI of 0, 10, 100, and 1000. LDH release was measured every 24 h post-infection up to 72 h. (**A**) Canine OS cell lines (D17, CF11, D22, and MC-KOS). (**B**) NCFs. These values are the average of three independent experiments.

In order to quantify and verify the cytotoxicity of CAV2-AU-M2 in the OS cell lines, we conducted lactate dehydrogenase (LDH) assays on all the canine OS cell lines and NCF cells. The cell cytotoxicity was determined by measuring the LDH released from the cells. Measurement of the LDH release (luminance) in the 2D culture every 24 h did not show a significant rise after infection with increased CAV2-AU-M2 MOI in the OS cells other than in D17 and CF11 (F-test *p*-value < 0.01; Figure 2A). There was a significant increase in the LDH release (luminance) in D17 and CF11 at 72 h at 1000 MOI (*t*-test *p*-values < 0.01; Supplementary Data). There was a barely significant increase in luminance over time for the NCFs with MOI of 10 (*t*-test *p*-value 0.045). The NCF cells at MOI of 100 and 1000, as well as D17 and CF11 cells at MOI of 0, 10, and 100 and MC-KOS at all MOI, showed no significant increased release of LDH (Figure 2A,B; Supplementary Data). Moreover, there was also a significant general increase in luminance over time for D22 (*t*-test *p*-value < 0.01) with or without the virus. Thus, the cytotoxicity in D22 would not appear to be a reflection of virus infection.

#### 3.1.2. CAV2-AU-M2 3D Cell Cultures

**Figure 3:**
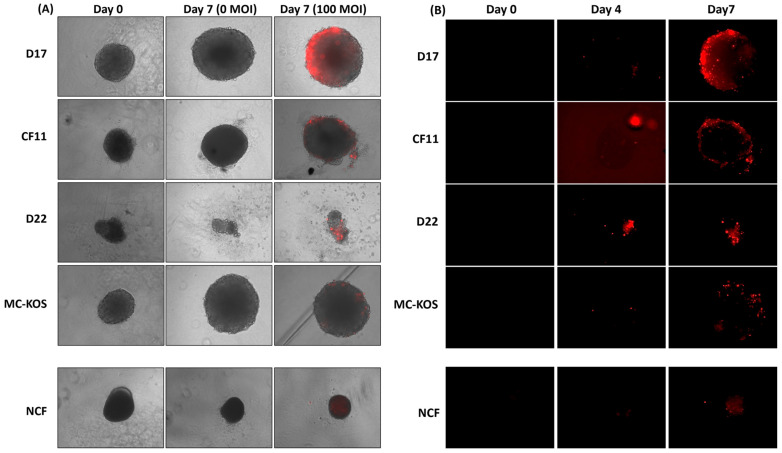
CAV2-AU-M2’s infectious and cytolytic properties in canine osteosarcoma cells in 3D culture. Spheroids were formed from all canine OS cell lines (D17, CF11, D22, and MC-KOS) and NCF cells. Spheroids were infected with CAV-AU-M2 at MOI of 100 and the DsRed fluorescent signal and spheroid shrinkage in cells was visualized using inverted fluorescent microscopy (Keyence) at 72 h post-infection at 10× magnification. (**A**) Size decrease and cytopathic effect comparison between infected and non-infected spheroids on day 7; (**B**) DsRed expression on day 0, 4, and 7 post-infection in spheroids. This is a representation of three independent experiments.

We investigated the efficiency of CAV2-AU-M2 infection and the lytic properties in the OS cell spheroids D17, CF11, D22 and MC-KOS and the NCF cells, which were incubated in 3D culture to mimic in vivo tumor growth patterns. This approach is thought to duplicate solid tumors by establishing geometry and three-dimensional cell–cell interactions. All the cell lines, except D22, and the NCF cells grew well as spheroids by 72 h. The infectivity and cytotoxic effects of CAV2-AU-M2 in the infected spheroids were monitored on day 0, day 4, and day 7 post-infection (Supplemental Figures S11–S20). On day 7 post-infection, the D17 and CF11 OS spheroids displayed minimal size shrinkage and compactness compared to the non-infected spheroids (0 MOI) (Figure 3A). In addition to a small decrease in size, there was some cellular debris in the peripheral circle in comparison to the non-infected spheroids, indicating cytotoxic effects. The outer surface of some infected spheroids exhibited variable irregularities and unevenness/a frail appearance, suggesting peripheral cell lysis (Figure 3A).

The infected spheroids exhibited an increasing amount of red fluorescence with increasing MOI and an increased duration post-infection (Figure 3B; Supplemental Figures S11–S20) in all the OS cell lines in comparison to the NCFs. The red fluorescence increased over time as the virus infected the OS spheroids in the outer layers and slowly diffused toward the core (Figure 3A,B). The virus infected the spheroids in the outer layers by 96 h (day 4) at 100 MOI and had slowly diffused toward the core by 7 days. On the other hand, the NCF spheroids were not significantly affected (Figure 3A,B). The NCF spheroids showed normal growth rates with minimal viral infection and replication (reduced spheroid size due to low replication) compared with the OS cells. By day 7, CAV2-AU-M2 had only infected the outermost shell in the NCF cells, with the remaining inner core cells showing no DsRed expression (Figure 3B).

Most of the control spheroids (MOI 0) grew larger over time, except D22 and the NCFs, which both shrank slightly. When the control spheroids were compared to infected spheroids of the same cells, we found that the only cell line for which the linear regression models were significant in explaining the size variability post-infection was D17 (F-test *p*-value < 0.01). The OS spheroids infected at MOI of 100 showed some evidence of cell death at day 4 and some size reduction by day 7 in D17 in comparison to the non-infected spheroids (*t*-test *p*-value 0.054; Table 1). At MOI of 1000, the spheroid size shrank significantly in D17 (*t*-test *p*-value < 0.01; Supplementary Data). The average spheroid size on day 7 (of infection) was 750 µm for MOI of 100 in D17, in comparison to 814 for MOI of 0. The CF11, D22, and MCKOS cell lines and the NCF cells did not show any significant difference in the size of the spheroids between the control and infected cells.

**Figure 4:**
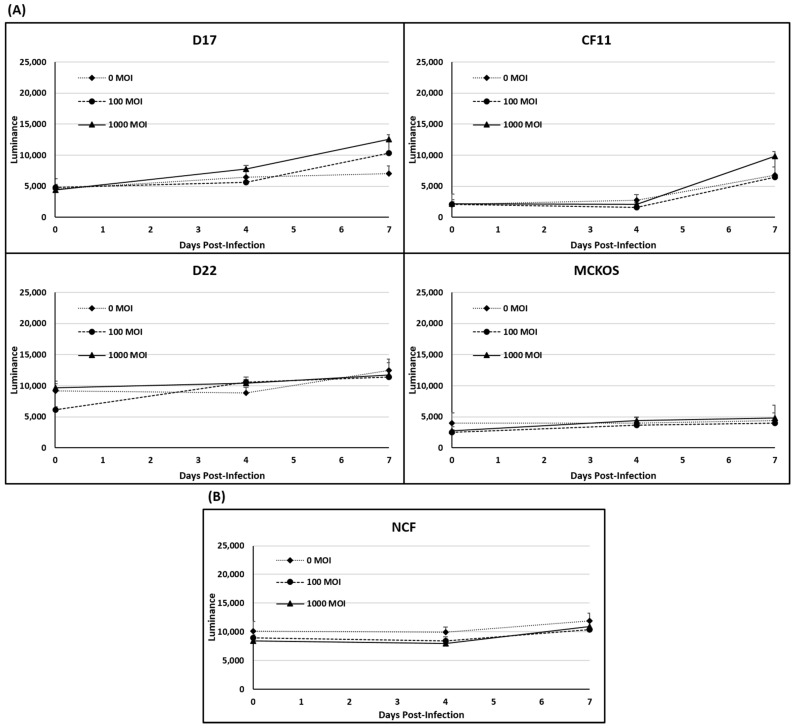
LDH release assay in 3D culture. (**A**) LDH release was measured in spheroids infected with CAV-AU-M2 at MOI of 0, 100, and 1000. LDH release was measured at 96 and 168 h post-infection. (**A**) Canine OS cell lines (D17, CF11, D22, and MC-KOS); (**B**) NCFs. These values are the average of three independent experiments.

The LDH release following CAV2-AU-M2 infection was elevated in the D17 and CF11 OS spheroids with increasing multiplicities of infection. CAV2-AU-M2 demonstrated higher cytotoxicity in the D17 OS cells at MOI of both 100 and 1000 and in the CF11 spheroids at MOI of 1000 in comparison to the non-infected spheroids (Figure 4A). A time-dependent increase in the LDH release was not observed in the D22, MC-KOS, and NCF cells post-infection (Figure 4A,B). However, when an F-test *p*-value < 0.01 was applied, there was no significant effect in any cell line.

### 3.2. Characterization of a His-Tagged Anti-PD-1 sdAb Produced in CAV2-AU-M2 Infected Cells

#### 3.2.1. Western Blot Analysis

**Figure 5:**
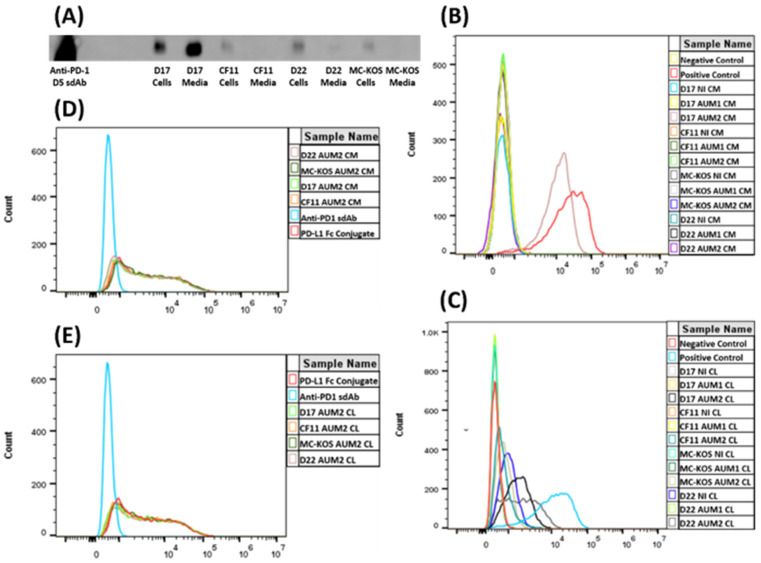
Functional properties of anti-PD-1 sdAb isolated from osteosarcoma cell lysates (CLs) and media infected with CAV2-AU-M2 virus. (**A**) Western blot analysis. Purified anti-PD-1 D5 sdAb was used as a positive control. Anti-PD-1 sdAb protein levels were measured via Western blotting using mouse anti-6x-His (Thermo) primary antibody and IRDye 800 CW goat anti-mouse (LI-COR) secondary antibody. (**B**) Flow cytometry analysis of anti-PD-1 sdAb binding to PD-1 receptor with cell count on the *y*-axis and fluorescence on the *x*-axis. Binding of anti-PD-1 sdAb purified from cell media collected from osteosarcoma cell lines (D17, CF11, MC-KOS, and D22) infected with CAV2-AU-M2 was compared with uninfected and CAV2-AU-M1-infected cells. (**C**) Flow cytometry analysis of anti-PD-1 sdAb binding to PD-1 receptor. Binding of anti-PD-1 sdAb purified from cell lysates collected from osteosarcoma cell lines (D17, CF11, MC-KOS, and D22) infected with CAV2-AU-M2 was compared with uninfected and CAV2-AU-M1-infected cells. (**D**) Flow cytometry analysis of PD-1/PD-L1 binding inhibition by anti-PD-1 sdAb. Inhibition of binding of PD-L1 to PD-1 by anti-PD-1 sdAb purified from cell media collected from osteosarcoma cell lines (D17, CF11, MC-KOS, and D22) infected with CAV2-AU-M2. (**E**) Flow cytometry analysis of PD-1/PD-L1 binding inhibition by anti-PD-1 sdAb. Inhibition of binding of PD-L1 to PD-1 by anti-PD-1 sdAb purified from cell lysates collected from osteosarcoma cell lines (D17, CF11, MC-KOS, and D22) infected with CAV2-AU-M2. CL, cell lysate; CM, cell media; NI, no infection.

The His-tagged anti-PD-1 sdAb production in the CAV2-AU-M2 infected cells was confirmed using Western blotting (Figure 5A). The same sdAb (anti-PD-1 D5), purified using HPLC (graciously gifted by Dr. Maninder Sandey), was used as a positive control. All the OS cell lines produced the anti-PD-1 sdAb (~15kb size) and released it into the media after cell lysis. D17 had the highest amount of the sdAb present in its medium due to having the most cell lysis (Figure 1B). There was a low amount of the anti-PD-1 sdAb present in the cell medium for D22 and none/minimal present in the media for CF11 and MC-KOS, but the sdAb was present in their cell lysates (Figure 5A).

#### 3.2.2. Assessment of Anti-PD-1 sdAb Binding to the PD-1 Receptors

Flow cytometry analysis was carried out to test the binding of the anti-PD-1 sdAb produced in the CAV2-AU-M2-infected cells with the PD-1 receptors expressed on the surface of HEK 293 cells. The antibodies purified from the cell lysates and media from the non-infected cells and the cells infected with the control virus CAV-AU-M1 showed low or minimal binding to PD-1 (Figure 5B,C; Table 2 and Table 3). The antibodies isolated from the cell lysates of the OS cells infected with CAV-AU-M2 displayed binding to the PD-1-expressing cells at varying ratios (11.95% to 64.48%; *p*-values < 0.01). The antibodies isolated from the D17 cell medium showed the highest binding to the PD-1-expressing cells, with 92.53% of cells bound (*p*-values < 0.01). The antibodies isolated from the CF11, D22, and MC-KOS cell media showed low/no binding to the cells expressing PD-1.

#### 3.2.3. Assessment of the Inhibition of PD-L1 Binding to PD-1

HEK 293 cells expressing PD-1 were used to assess the functional antagonist activity, in terms of PD-L1 binding, of the anti-PD-1 sdAbs produced by the CAV2-AU-M2-infected cells. Flow cytometry analysis using fluorescently tagged PD-L1 revealed that, in addition to binding to PD-1, the CAV-AU-M2-produced anti-PD-1 sdAb may also inhibit the binding of PD-L1 to PD-1, as shown by the decreasing percentage of PD-L1-labeled cells (Figure 5D,E; Table 4). In the absence of the anti-PD-1 sdAb, 76.10% of cells were stained by the AF647-labeled canine Fc-conjugated human PD-L1 protein. The anti-PD-1 sdAb isolated from the OS cell lines infected with CAV-AU-M2 significantly (*p*-values < 0.01) inhibited the PD-1/PD-L1 interaction, as reflected by the decrease in the cells labeled by PD-L1 (71.04%, D17 cell media; 72.66%, D17 cell lysate; 72.25%, D22 cell media; 74.01%, D22 cell lysate; and 73.97, CF11 cell lysate). The anti-PD-1 sdAb isolated from the CF11 cell media and MC-KOS cell media and lysates did not inhibit PD-L1 binding with the PD-1 receptor (Table 4; Figure 5D,E).

## 4. Discussion

The low “5-year” survival rate of metastatic osteosarcoma is suggestive of the limited effectiveness of the current treatment options, as they only provide benefits to a small number of patients [26]. The preclinical and clinical findings show that oncolytic vectors can trigger anti-tumor immunity and increase the immune cell infiltration (tumor neo-antigen presentation and enhanced infiltration and cytotoxic activity of the T cells) into the tumor microenvironment (TME) [27,28]. This can switch an immunologically “cold” TME into a “hot” one in terms of the innate and adaptive immune cell infiltration in the TME [29]. The induced adaptive immune response targets the primary tumor site and distant tumor metastases that were not exposed to the virus in a response known as the bystander effect [30,31,32,33,34,35,36,37,38,39,40].

Oncolytic vectors encoding genes for immunomodulatory proteins have the potential to widen the applications of cancer immunotherapy, including in osteosarcoma treatment [37,38,39,41]. A range of genes have already been integrated into oncolytic adenoviruses to augment immune stimulation, including T cell priming and antigen presentation [42].

We have designed a next-generation CRAd, CAV2-AU-M2, armed with an anti-PD-1 single-domain antibody (sdAb) [24]. Our CRAd is designed to lyse only tumor cells and deliver an anti-PD-1 sdAb locally to the TME to avoid systemic adverse effects and stimulate anti-tumor innate and adaptive immune responses. Recombinant adenoviruses are limited to a packaged genome size of 105% of the wild-type adenovirus [43]. Therefore, in CAV2-AU-M2, the E3 gene was replaced with an anti-PD-1 sdAb sequence to keep the size under 105% of the wild type. This replacement served to keep the size of virus genome within the limit and to remove the E3 gene’s negative effect on anti-tumor immunity. E3 encodes for an immunomodulatory protein that protects virus-infected cells from being killed by cytotoxic T cells and death-inducing cytokines [44]. E3 also encodes for proteins that inhibit TNF-induced apoptosis [44]. The anti-PD-1 sdAb sequence inserted into CAV2-AU-M2 does not have a secretory signal encoded in it. The intention behind this is to restrict the anti-PD-1 sdAb release to cell lysis post-viral infection. With this design in mind, the anti-PD-1 sdAb will be produced in any infected cells (OS or normal) but will only be released into the TME due to the cell lysis induced by CAV2-AU-M2 infection. The virus was designed in this way to avoid systemic release.

In our previous findings, CAV2-AU-M2 showed the ability to infect and replicate in multiple tumor cell lines (osteosarcoma and breast tumor) [24]. In this manuscript, we have expanded our findings in terms of the cytotoxic and replicative properties of CAV2-AU-M2 and documented its ability to produce and release an anti-PD-1 sdAb into the TME. Our data showed that CAV2-AU-M2 demonstrated a noticeable decrease in cell confluence in two OS cell lines due to the induction of cytotoxic processes and an increase in virus spread and infection (Figure 1, Figure 2, Figure 3 and Figure 4). Our virus did not show cytotoxicity in D22 and MC-KOS, but the infection clearly spread over time and with an increased viral dosage. CAV2-AU-M2 showed limited initial infection and did not show viral spread or cell lysis in normal cells. Collectively, these findings highlight the profound impact of CAV2-AU-M2 as an oncolytic adenovirus on the morphology of OS cell cultures, suggesting the initiation of cytotoxic processes and apoptotic cell death pathways.

Fluorescent microscopic images indicated that CAV2-AU-M2 infected, replicated in, and lysed D17 and CF11 osteosarcoma cell lines in 2D and 3D cultures. Importantly, the cytopathic effects were specific to more prolific OS cells, as the less prolific cell lines D22 and MC-KOS and the non-cancerous cells, NCFs, did not show active CPE, highlighting the replication selectivity and safety of CAV2-AU-M2 in non-cancerous cells (Figure 1 and Figure 2). The D17 and CF11 OS cells infected with CAV2-AU-M2 revealed cell shrinkage and increased intercellular space, which could be attributed to the cellular stress or apoptotic processes induced by this oncolytic adenovirus. This reduction in cell size was accompanied by a disruption in the normal cell adherence pattern, characterized by reduced or altered cell–cell contact.

We further investigated the efficiency of CAV2-AU-M2 infection and its lytic properties in OS cell spheroids. The spheroid-based model represents tumor nodules and micro-metastases and demonstrates the ability of the virus to move through denser tumor masses. Our results demonstrated that CAV2-AU-M2 effectively infected and lysed the D17 OS spheroids in a time- and dose-dependent manner (Figure 4; Supplementary Figures S11–S20). The infected spheroids displayed size shrinkage (Table 1), increased cellular debris, and variable irregularities on their outer surface, indicating the cytotoxic effects of CAV2-AU-M2. These findings demonstrate the ability of CAV2-AU-M2 as an oncolytic adenovirus to selectively penetrate, replicate, and exert its cytotoxic effects within the complex three-dimensional architecture of OS spheroids, highlighting its potential as a therapeutic strategy against these tumors in vivo. However, the CF11, D22, and MC-KOS OS cells showed an increase in virus infection but not lysis. A higher dosage and increased time may be required to exhibit the same effect in CF11, D22, and MC-KOS.

The LDH cytotoxicity assay data also indicated increased LDH release in the D17 and CF11 OS cells infected with CAV2-AU-M2 compared to the non-infected cells and the D22, MC-KOS, and NCF cells. The release of LDH from the infected cells indicates cell damage and cytolysis. This finding further supports the potent cytotoxic effects of CAV2-AU-M2 on the D17 and CF11 OS cells. This elevated LDH release suggests a substantial loss of cell membrane integrity and the release of the LDH enzyme from the cytosol into the culture medium, indicating cell death. These results demonstrate that oncolytic adenovirus infection induces cytotoxic effects in osteosarcoma cell cultures.

The adenoviral E3 gene also encodes the Ad death protein (ADP) in large amounts at the very late stages of infection [45]. ADP is responsible for effective cell lysis of Ad-infected cells to release virus progeny [45]. CAV2-AU-M2 has an E3 deletion, which may be responsible for its less efficient cell lysis in D22 and MC-KOS OS cells. D17 and CF11 likely showed a higher replication and lysis due to the viral burden. While D22 and MC-KOS did show increased virus replication (increased red fluorescence), the virus progeny burden was likely not enough to induce cell lysis. Meanwhile, the NCFs showed low or minimal virus replication and no cell lysis. This varied effect underscores the potential need for an improved oncolytic virus design with an intact E3 gene to better lyse tumor cells that may be less efficient at replicating the virus.

In combination with checkpoint inhibitors, oncolytic viruses have a more profound effect on tumors [38,46,47,48]. PD-1 is an immune checkpoint protein that keeps the T cells from attacking the tumor cells by inactivating them [49,50,51,52]. Monoclonal antibodies (mAbs) against immune checkpoints such as PD-1 activate the immune system in the TME. An immune checkpoint blockade is more successful in immunologically “hot” tumors. Therefore, strategies to convert a cold TME into a hot TME will improve the outcomes of immune checkpoint blockade therapy. Oncolytic virotherapy improves anti-PD-1 immunotherapy by promoting intertumoral cell infiltration [53]. CRAd vectors designed to produce anti-PD-1 antibodies directly in the TME boost the immune system and enable the T cells to kill tumor cells. Tumor-localized secretion of the soluble PD-1 protein enhances oncolytic virotherapy and avoids systemically adverse effects [48].

CAV2-AU-M2 is a next-generation CRAd that produces an anti-PD-1 sdAb in tumor cells. We characterized the anti-PD-1 sdAb production and its functional properties with respect to binding PD-1 and inhibiting PD-L1 binding to PD-1. All the osteosarcoma cell lines (D17, CF11, D22, and MCKOS) infected with CAV2-AU-M2 were expected to produce an anti-PD-1 sdAb of approximately 15 KD. There is no signal peptide preceding the sdAb; therefore, the anti-PD-1 sdAb was released only after cell lysis. Production of the anti-PD-1 sdAb by CAV2-AU-M2 was confirmed using Western blotting (Figure 5A). The anti-PD-1 sdAb was present in the cell lysates from all the OS cell lines. However, there was low or minimal release in the cell media of CF11 and MCKOS post-virus infection. The antibody was purified post 48 h of infection, and CF11 and MCKOS had lower cell lysis in comparison to D17 and D22 (Supplementary Figures S1–S10). It may also be possible that due to the absence of a secretory signal, the antibody was concentrated mainly in the cells and was not adequately released into the cell media after lysis. Given the lower levels of lysis in CF11 and MCKOS, this would explain the low levels of the anti-PD-1 sdAb in the media.

In order to confirm the potential of the secreted anti-PD-1 sdAb to bind with its ligand canine PD-1 receptor, we performed flow cytometry. HEK 293 cells expressing the PD-1 receptor were incubated with the anti-PD-1 sdAbs isolated from the media and cell lysates from the CAV2-AU-M1- and CAV2-AU-M2-infected osteosarcoma cell lines. A shift to the right in the fluorescence in the histogram of the PD-1+ HEK 293 cells compared to that of the negative controls shows the anti-PD-1 sdAb binding with the canine PD-1. Binding was observed only in the cells incubated with the media (D17) and cell lysates (D17, CF11, D22, and MCKOS) from the cells infected by CAV2-AU-M2 (Figure 5B,C; Table 2) in comparison to with the media and cell lysates from the cells infected by CAV2-AU-M1. With this experiment, we have demonstrated that our virus vector produces an anti-PD-1 sdAb that can bind to the PD-1 receptor on the cells and that the anti-PD-1 sdAb is released into cell culture media after cell lysis.

Another important aspect of our CRAd is to ensure that the anti-PD-1 sdAb release will inhibit the PD-1/PD-L1 interaction in order to boost the T cell immunity in the TME. We tested the efficacy of the competitive binding of the anti-PD-1 sdAb by assaying the ability of PD-L1 to bind to PD-1. The HEK 293 cells expressing PD-1 were incubated with Fc-conjugated PD-L1 with or without the presence of the anti-PD-1 sdAb. The anti-PD-1 sdAb isolated from the D17 media and cell lysates and D22 media minimally inhibited the binding of PD-L1 to the PD-1 receptor (Figure 5D and Table 3), as evidenced by the lower number of cells labeled by the PD-L1 (71.04 and 72.06%, respectively). However, the anti-PD-1 sdAb samples isolated from all the other OS media and cell lysates did not inhibit PD-L1 binding with PD-1. This was most likely a concentration-dependent effect due to ineffective cell lysis and failing to release enough of the anti-PD-1 sdAb to inhibit PD-1–PD-L1 binding.

Our collective data show that we have successfully designed and developed a next-generation CRAd armed with a functional anti-PD-1 sdAb. Our CRAd will be able to stimulate an anti-tumor immune response in the TME owing it its oncolytic properties, including inhibiting the immune checkpoint protein PD-1 from binding to its ligand, PD-L1. Our CRAd signifies the combinatorial immunotherapy approach for “hard-to-treat” cancer treatment, such as osteosarcoma. Our virus can enhance the immunity in the TME by stimulating T cell infiltration and recruiting the T cells against the tumor cells. Thus, our virus synergizes the advantage of both immunotherapies and overcomes their setbacks in treating osteosarcoma. The virus production of the anti-PD-1 sdAb locally in the TME will also reduce the adverse systemic effects of traditional anti-PD-1 therapy. However, as stated earlier, due to E3 deletion and the lack of a secretory signal in the anti-PD-1 sdAb, CAV2-AU-M2 has some limitations in its efficiency.

The results reported, however, do support our theory of a next-generation tumor-specific oncolytic virus that can produce an anti-PD-1 sdAb in the TME. Owing to this function, our oncolytic virus can stimulate an immune response in TME due to virus infection, along with the inhibition of PD-1–PD-L1 binding.

## 5. Conclusions

We were successful in making the next-generation conditionally replicative virus CAV2-AU-M2. This virus will have a synergistic effect in inducing immunity against tumor cells in the tumor microenvironment according to oncolysis and by inhibiting the immune checkpoint PD-1.

## Figures and Tables

**Table 1:** Canine OS cell line (D17, CF11, D22, and MC-KOS) spheroid size on day 0 and 7 at 0 and 100 MOI of CAV2-AU-M2.

Cell Line	MOI	Day 0 (µm)	Day 7 (µm)
D17	0	517	814
	100	551	750
CF11	0	401	563
	100	400	507
D22	0	339	264
	100	425	317
MC-KOS	0	539	601
	100	459	657
NCF	0	455	386
	100	447	399

These sizes are the average spheroid sizes from three independent experiments.

**Table 2:** Flow cytometry analysis of binding of anti-PD-1 sdAb to PD-1-receptor-expressing cells. Anti-PD-1 sdAb was purified using NTA columns from the cell media of non-infected cells and cells infected by CAV2-AU-M1 and CAV2-AU-M2. APC-labeled anti-His mouse primary antibody was used to visualize sdAb bound to cells.

Cell Line	Cell Treatment	% of Cells Labeled by Anti-PD-1 sdAb	Standard Deviation
	Purified anti-PD-1 D5 sdAb (positive control)	94.26	0.90
D17	Non-infected	0.12	0.02
	CAV-AU-M1	0.09	0.03
	CAV-AU-M2	92.53	3.66
CF11	Non-infected	0.17	0.03
	CAV-AU-M1	0.09	0.04
	CAV-AU-M2	0.51	0.09
MC-KOS	Non-infected	0.66	0.11
	CAV-AU-M1	0.56	0.12
	CAV-AU-M2	0.55	0.07
D22	Non-infected	0.60	0.08
	D22 CAV-AU-M1	0.64	0.08
	D22 CAV-AU-M2	0.36	0.11

This table is the average of three independent experiments.

**Table 3:** Flow cytometry analysis of binding of anti-PD-1 sdAb to PD-1-receptor-expressing cells. Anti-PD-1 sdAb was purified using NTA columns from cell lysates of non-infected cells and cells infected by CAV2-AU-M1 and CAV2-AU-M2. APC-labeled anti-His mouse primary antibody was used to visualize sdAb bound to cells.

Cell Line	Cell Treatment	% of Cells Labeled by Anti-PD-1 sdAb	Standard Deviation
	Purified anti-PD-1 D5 sdAb (positive control)	94.26	0.90
D17	Non-infected	0.10	0.03
	CAV-AU-M1	0.06	0.02
	CAV-AU-M2	64.48	1.72
CF11	Non-infected	0.09	0.06
	CAV-AU-M1	0.11	0.06
	CAV-AU-M2	11.95	2.34
MC-KOS	Non-infected	0.68	0.03
	CAV-AU-M1	0.51	0.06
	CAV-AU-M2	12.90	4.99
D22	Non-infected	0.14	0.05
	D22 CAV-AU-M1	0.63	0.07
	D22 CAV-AU-M2	52.95	4.11

This table is the average of three independent experiments.

**Table 4:** Flow cytometry analysis of competitive inhibition of PD-1/PD-L1 binding by anti-PD-1 sdAb. Anti-PD-1 sdAb was purified using NTA columns from cell media and lysates of cells infected by CAV2-AU-M2. APC-labeled canine Fc-conjugated human PD-L1 protein was used to label the PD-1 receptor.

Cell Line	Source of Anti-PD-1 sdAb	% of Cells Labeled by Anti-AF647-Labeled PD-L1 Protein
	No Anti-PD-1 sdAb	76.10
	Purified Anti-PD-1 D5 sdAb	2.52
D17	Cell Media	71.04
	Cell Lysate	72.66
CF11	Cell Media	76.35
	Cell Lysate	73.97
MC-KOS	Cell Media	80.72
	Cell Lysate	77.35
D22	Cell Media	72.25
	Cell Lysate	74.01

This table is the average of three independent experiments.

## Data Availability

All the data and material that support the findings of this study are included in this manuscript and the Supplementary Materials.

## Supplementary Materials

The following supporting information can be downloaded at: https://www.mdpi.com/article/10.3390/cells13040351/s1, Figure S1: Red fluorescent images of CAV2-AU-M2 infections in D17 cell line at different MOIs and time point; Figure S2: Red fluorescent images of CAV2-AU-M2 infections in CF11 cell line at different MOIs and time point; Figure S3: Red fluorescent images of CAV2-AU-M2 infections in D22 cell line at different MOIs and time point; Figure S4: Red fluorescent images of CAV2-AU-M2 infections in MCKOS cell line at different MOIs and time point; Figure S5: Red fluorescent images of CAV2-AU-M2 infections in NCF at different MOIs and time point; Figure S6: Phase contrast images of CAV2-AU-M2 infections in D17 cell line at different MOIs and time point ; Figure S7: Phase contrast images of CAV2-AU-M2 infections in CF11 cell line at different MOIs and time point; Figure S8: Phase contrast images of CAV2-AU-M2 infections in D22 cell line at different MOIs and time point; Figure S9: Phase contrast images of CAV2-AU-M2 infections in MCKOS cell line at different MOIs and time point; Figure S10: Phase contrast images of CAV2-AU-M2 infections in NCF at different MOIs and time point; Figure S11: Red fluorescent images of CAV2-AU-M2 infections in D17 cell line spheroids at different MOIs and time point; Figure S12: Phase contrast images of CAV2-AU-M2 infections in D17 cell line spheroids at different MOIs and time point; Figure S13: Red fluorescent images of CAV2-AU-M2 infections in CF11 cell line spheroids at different MOIs and time point; Figure S14: Phase contrast images of CAV2-AU-M2 infections in CF11 cell line spheroids at different MOIs and time point; Figure S15: Red fluorescent images of CAV2-AU-M2 infections in D22 cell line spheroids at different MOIs and time point; Figure S16: Phase contrast images of CAV2-AU-M2 infections in D22 cell line spheroids at different MOIs and time point; Figure S17: Red fluorescent images of CAV2-AU-M2 infections in MCKOS cell line spheroids at different MOIs and time point; Figure S18: Phase contrast images of CAV2-AU-M2 infections in MCKOS cell line spheroids at different MOIs and time point; Figure S19: Red fluorescent images of CAV2-AU-M2 infections in NCF spheroids at different MOIs and time point; Figure S20: Phase contrast images of CAV2-AU-M2 infections in NCF spheroids at different MOIs and time point; Data: Linear regression for size and luminance
