# Supplementary material for: The Development and Characterization of a Next-Generation Oncolytic Virus Armed with an Anti-PD-1 sdAb for Osteosarcoma Treatment In Vitro
Source: Cells. 2024 Feb 17;13(4):351. doi: 10.3390/cells13040351 (PMC10886739; doi:10.3390/cells13040351)
Supplement: Supplementary file 1 [file cells-13-00351-s001.zip › cells-2802244-supplementary.pdf]

Abbreviation	Abbreviation Meaning
CAV2	Canine adenovirus type 2
OS	Osteosarcoma
CRAd	Conditionally Replicative Adenovirus
sdAb	single-domain Antibody
PD-1	programmed cell death protein 1
PD-L1	programmed death-ligand 1
PD-L2	programmed death-ligand 2
TME	Tumor Microenvironment
mAb	Monoclonal antibody
CPE	Cytopathic effects
NCF	Normal Canine Fibroblasts
MOI	multiplicity of infection
LDH	lactate dehydrogenase

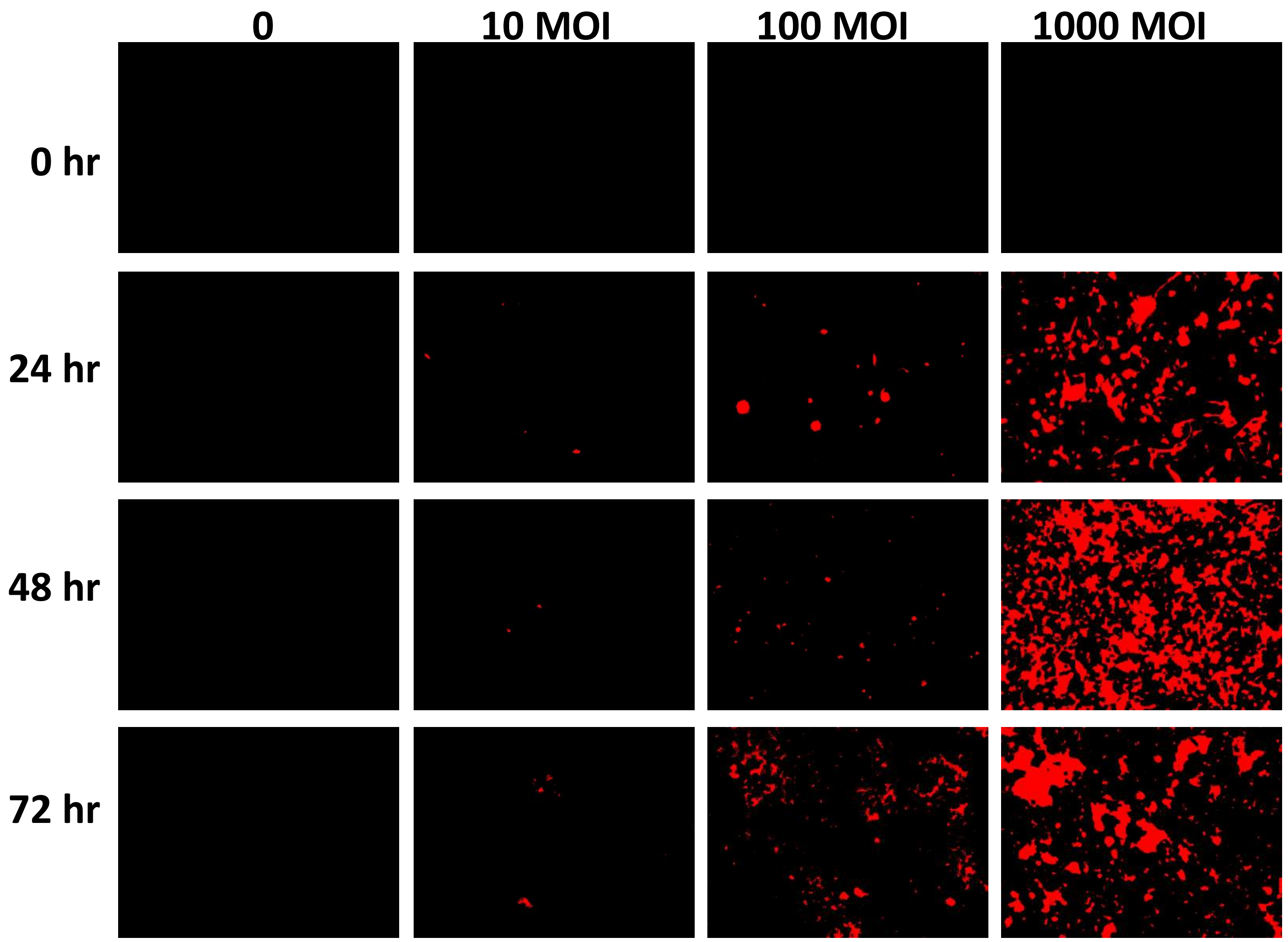

Figure S1: Red fluorescent images of CAV2-AU-M2 infections in D17 cell line at different MOIs and time point. D17 cells were infected with CAV-AUM2 at MOI 0, 10,100, and 1000, and the dsRed fluorescent signal was visualized at 0 h, 24 h, 48 h and 72 h post-infection by fluorescence microcopy (Keyence) at 10X magnification These is a representation of three independent experiments.

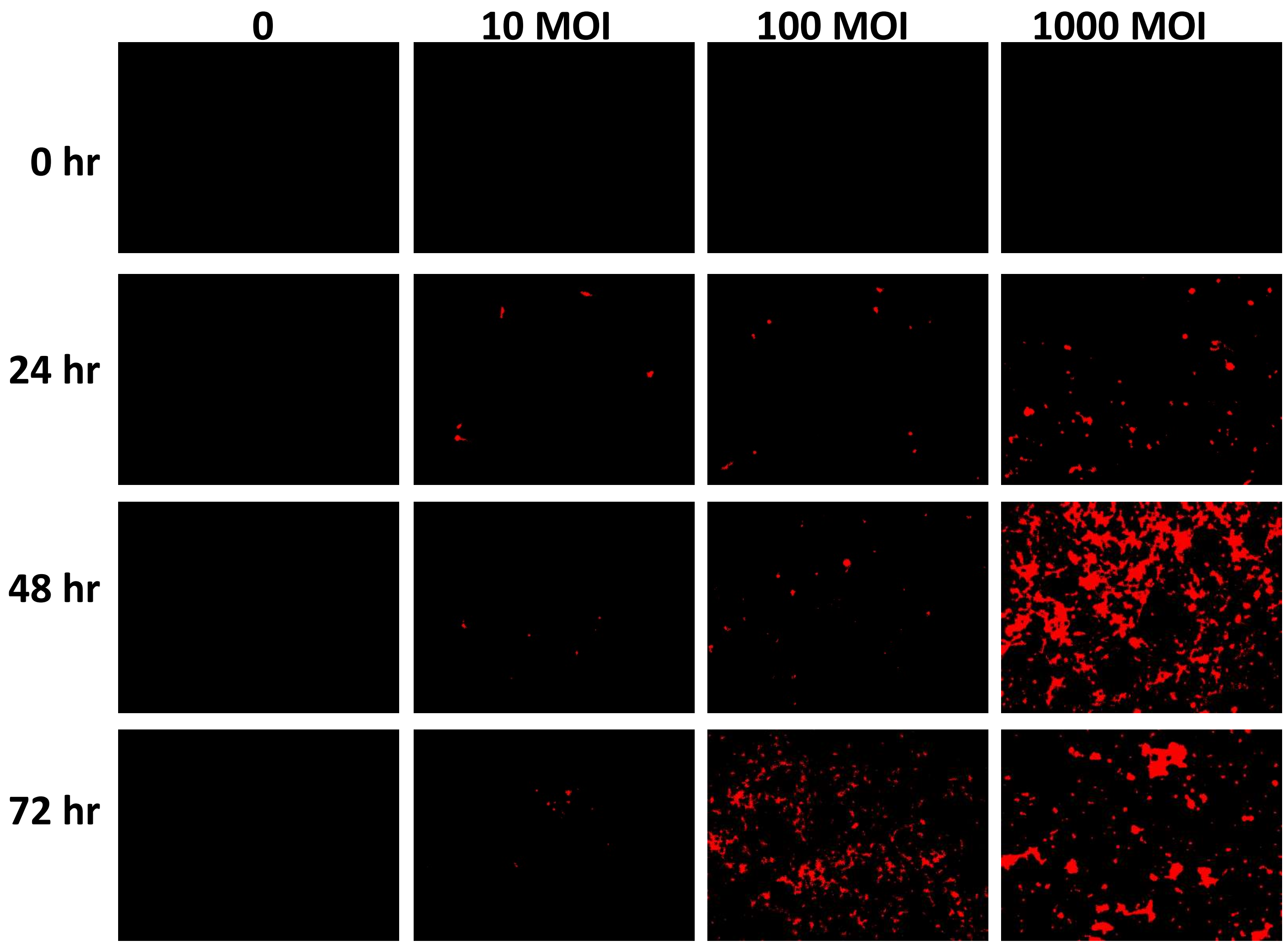

Figure S2: Red fluorescent images of CAV2-AU-M2 infections in CF11 cell line at different MOIs and time point. CF11cells were infected with CAV-AUM2 at MOI 0, 10,100, and 1000, and the dsRed fluorescent signal was visualized at 0 h, 24 h, 48 h and 72 h post-infection by fluorescence microcopy (Keyence) at 10X magnification These is a representation of three independent experiments.

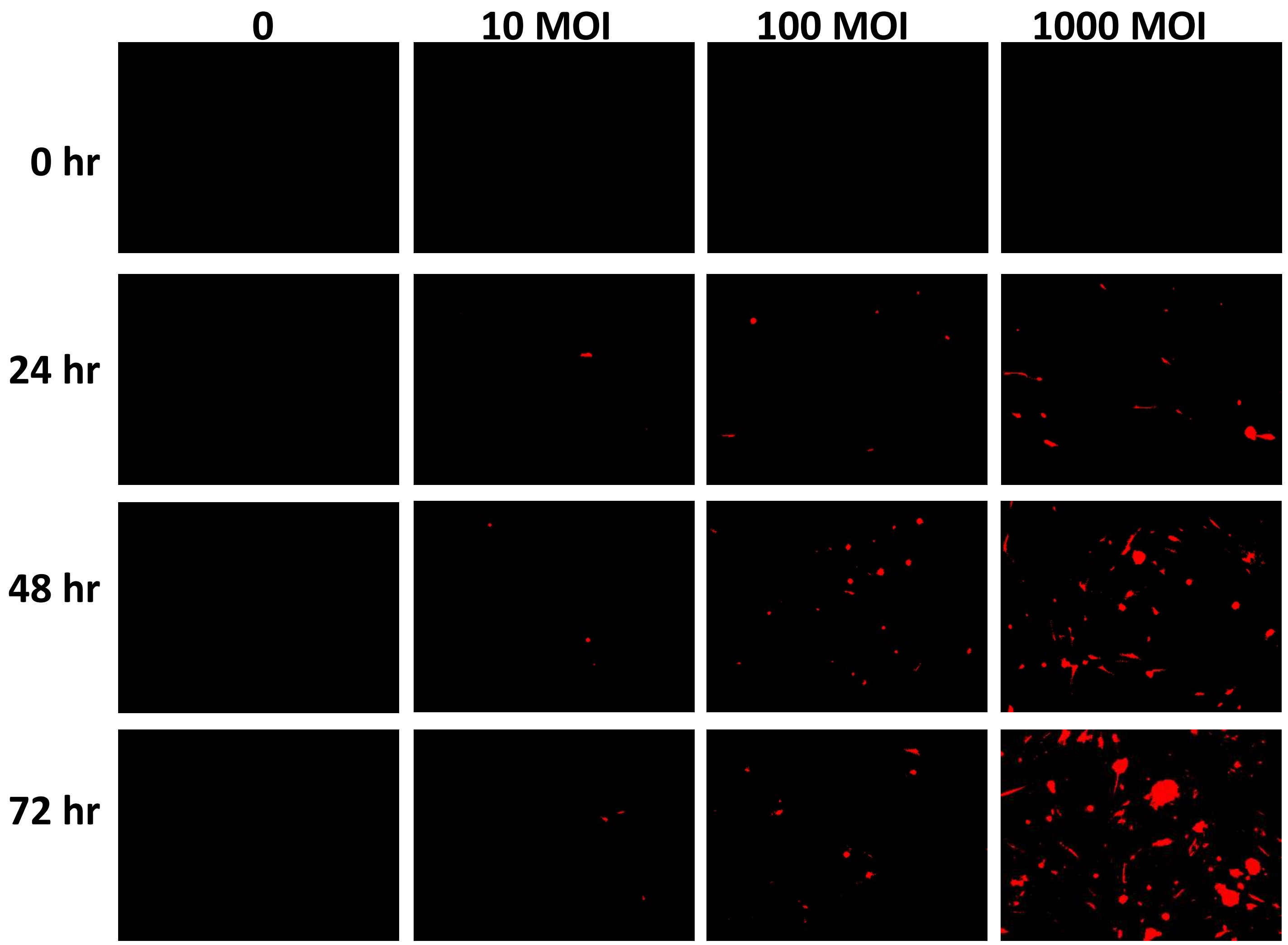

Figure S3: Red fluorescent images of CAV2-AU-M2 infections in D22 cell line at different MOIs and time point. D22 cells were infected with CAV-AUM2 at MOI 0, 10,100, and 1000, and the dsRed fluorescent signal was visualized at 0 h, 24 h, 48 h and 72 h post-infection by fluorescence microcopy (Keyence) at 10X magnification These is a representation of three independent experiments.

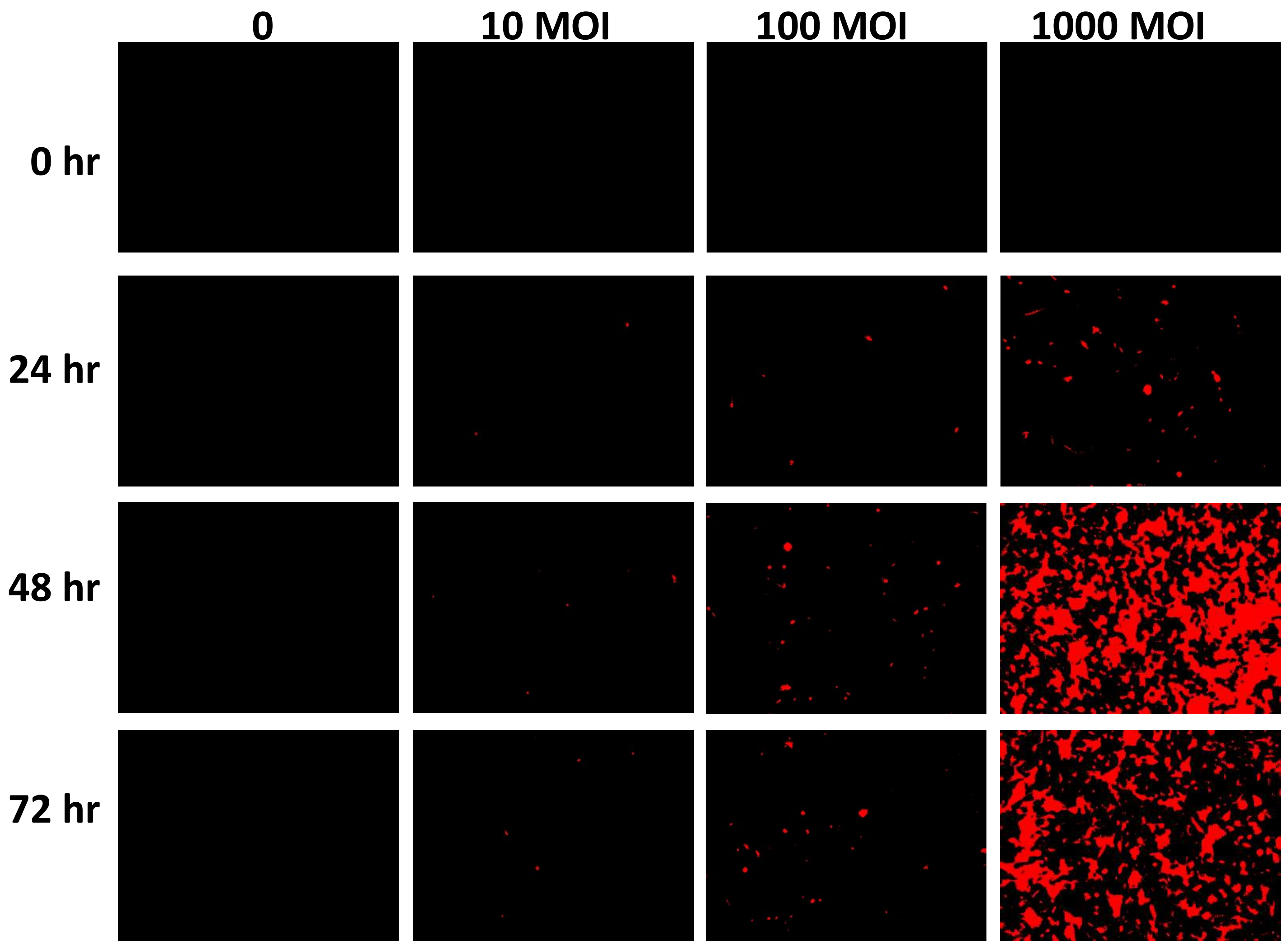

Figure S4: Red fluorescent images of CAV2-AU-M2 infections in MCKOS cell line at different MOIs and time point. MCKOS cells were infected with CAV-AUM2 at MOI 0, 10,100, and 1000, and the dsRed fluorescent signal was visualized at 0 h, 24 h, 48 h and 72 h post-infection by fluorescence microcopy (Keyence) at 10X magnification These is a representation of three independent experiments.

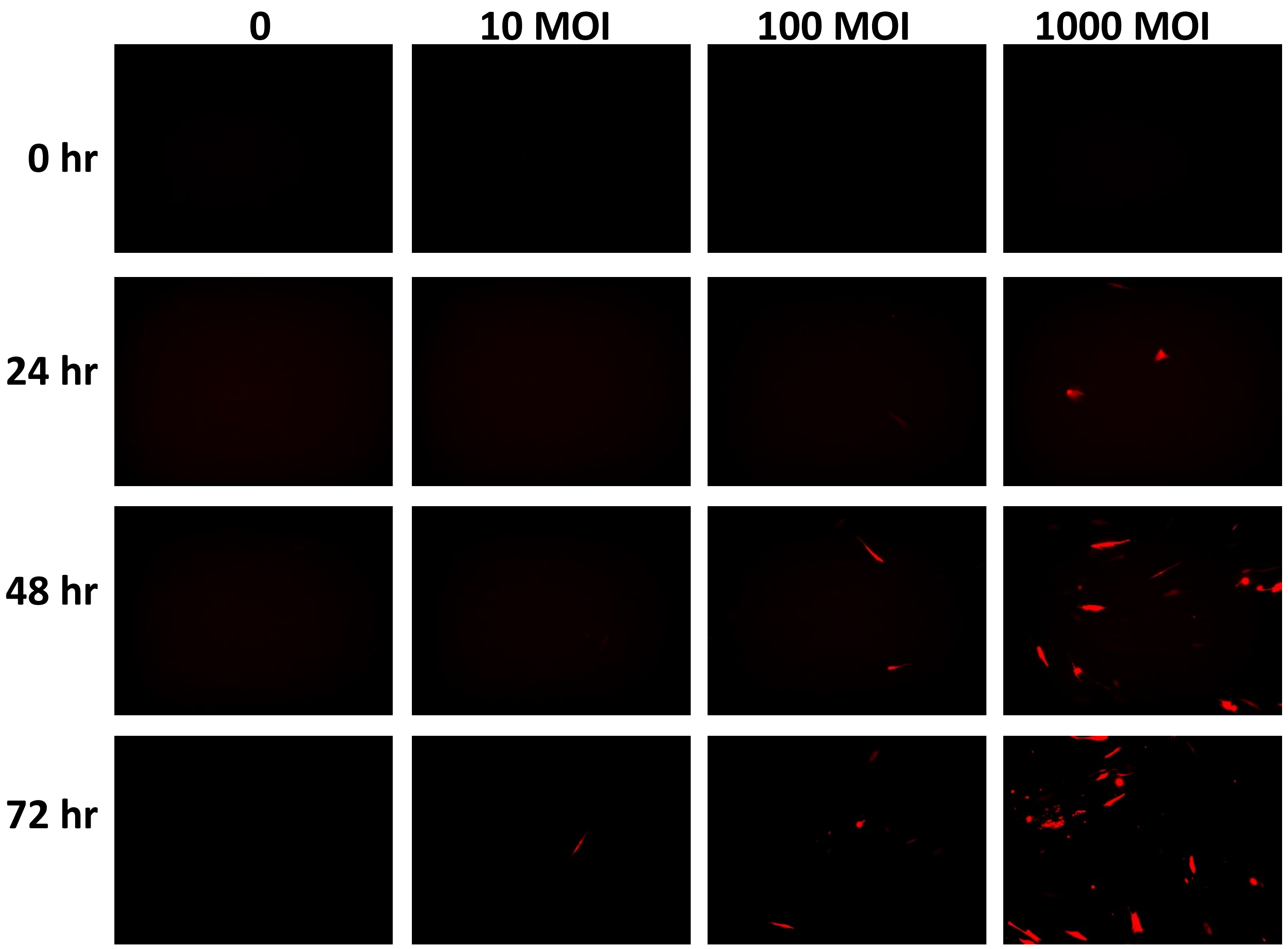

Figure S5: Red fluorescent images of CAV2-AU-M2 infections in NCF at different MOIs and time point. NCF cells were infected with CAV-AUM2 at MOI 0, 10,100, and 1000, and the dsRed fluorescent signal was visualized at 0 h, 24 h, 48 h and 72 h post-infection by fluorescence microcopy (Keyence) at 10X magnification These is a representation of three independent experiments.

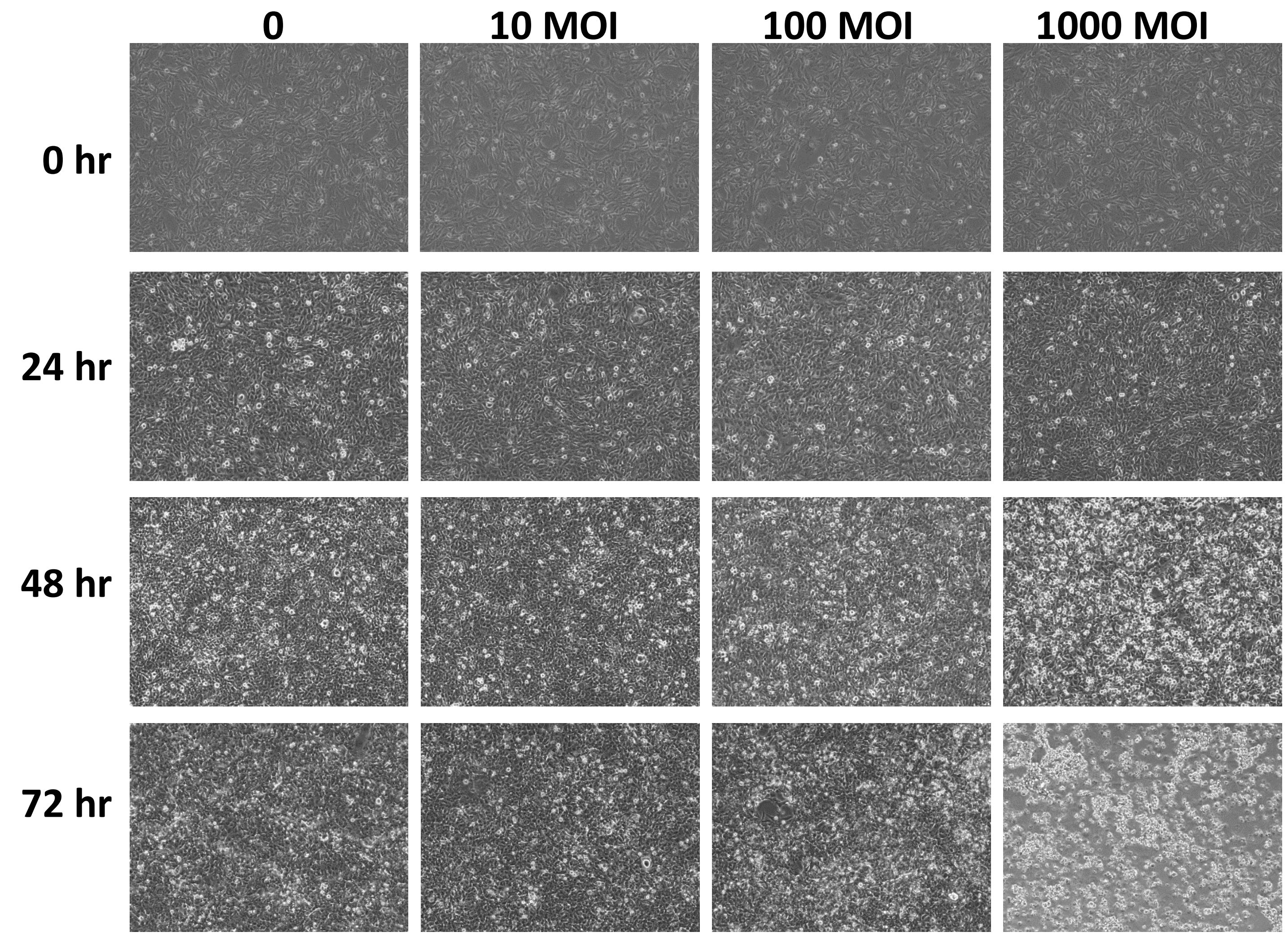

Figure S6: Phase contrast images of CAV2-AU-M2 infections in D17 cell line at different MOIs and time point. D17 cells were infected with CAV-AUM2 at MOI 0, 10,100, and 1000, and the cytopathic effect was visualized at 0 h, 24 h, 48 h and 72 h post-infection by fluorescence microcopy (Keyence) at 10X magnification These is a representation of three independent experiments.

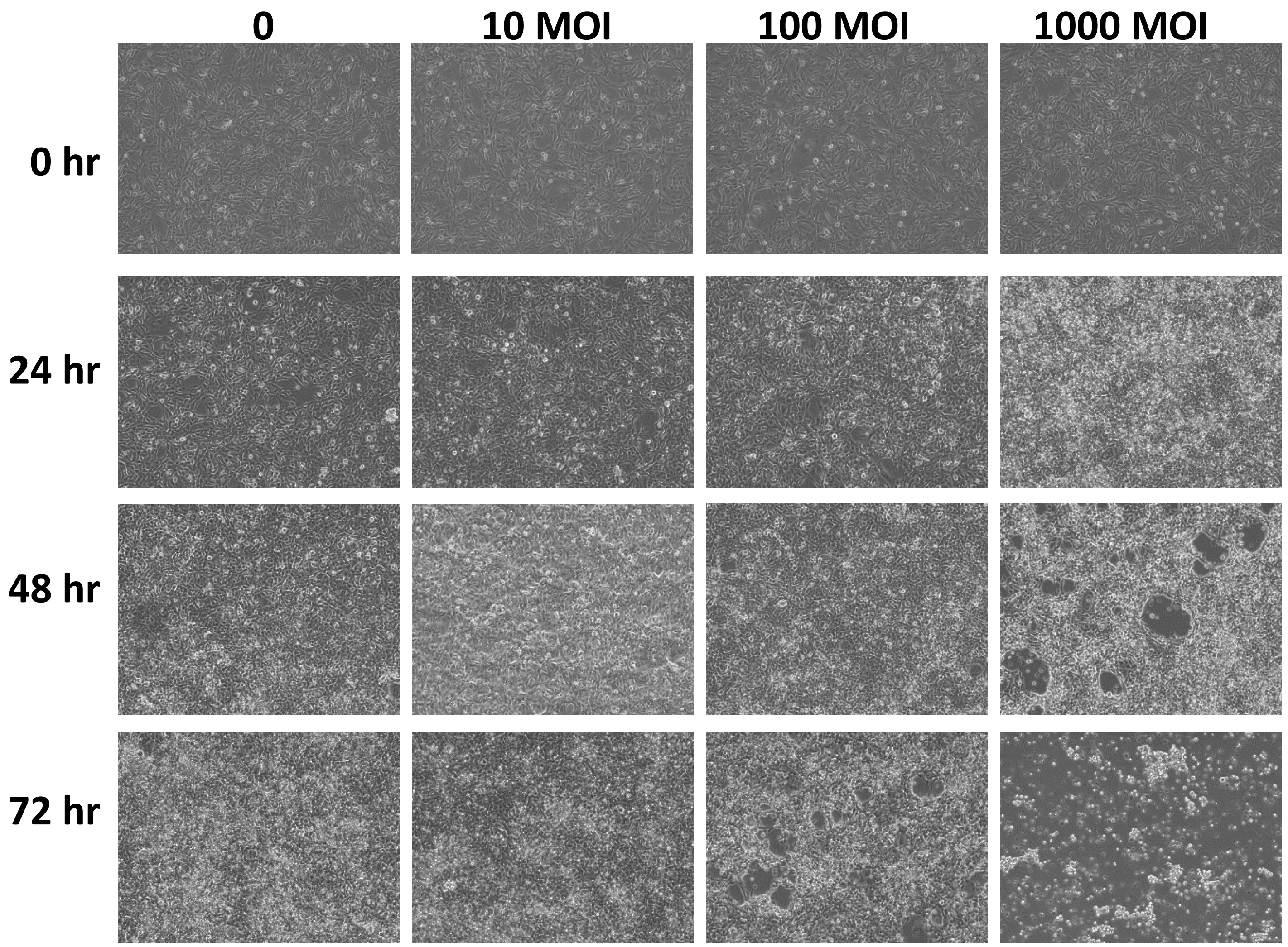

Figure S7: Phase contrast images of CAV2-AU-M2 infections in CF11 cell line at different MOIs and time point. CF11 cells were infected with CAV-AUM2 at MOI 0, 10,100, and 1000, and the cytopathic effect was visualized at 0 h, 24 h, 48 h and 72 h post-infection by fluorescence microcopy (Keyence) at 10X magnification These is a representation of three independent experiments.

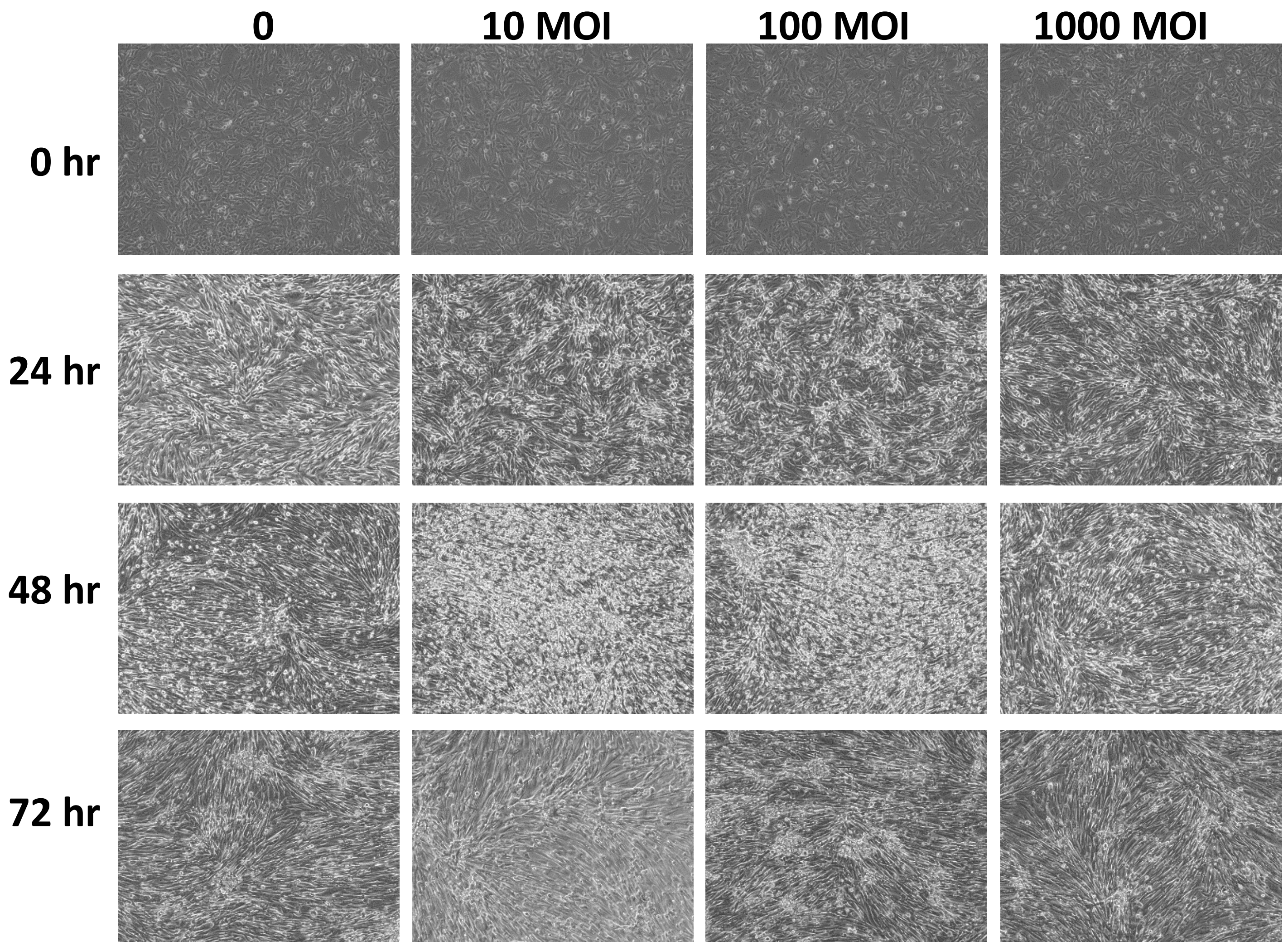

Figure S8: Phase contrast images of CAV2-AU-M2 infections in D22 cell line at different MOIs and time point. D22 cells were infected with CAV-AUM2 at MOI 0, 10,100, and 1000, and the cytopathic effect was visualized at 0 h, 24 h, 48 h and 72 h post-infection by fluorescence microcopy (Keyence) at 10X magnification These is a representation of three independent experiments.

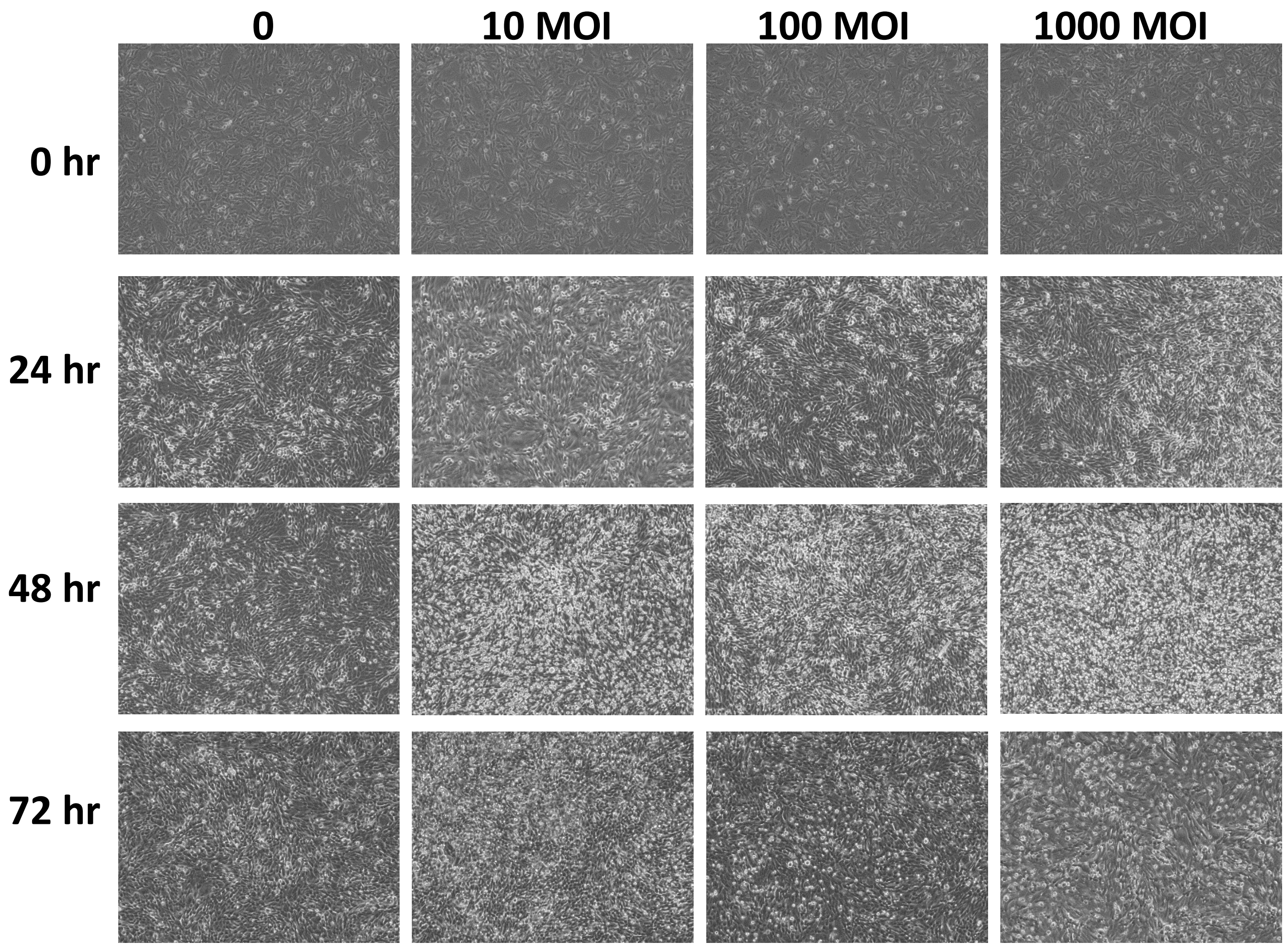

Figure S9: Phase contrast images of CAV2-AU-M2 infections in MCKOS cell line at different MOIs and time point. MCKOS cells were infected with CAV-AUM2 at MOI 0, 10,100, and 1000, and the cytopathic effect was visualized at 0 h, 24 h, 48 h and 72 h post-infection by fluorescence microcopy (Keyence) at 10X magnification These is a representation of three independent experiments.

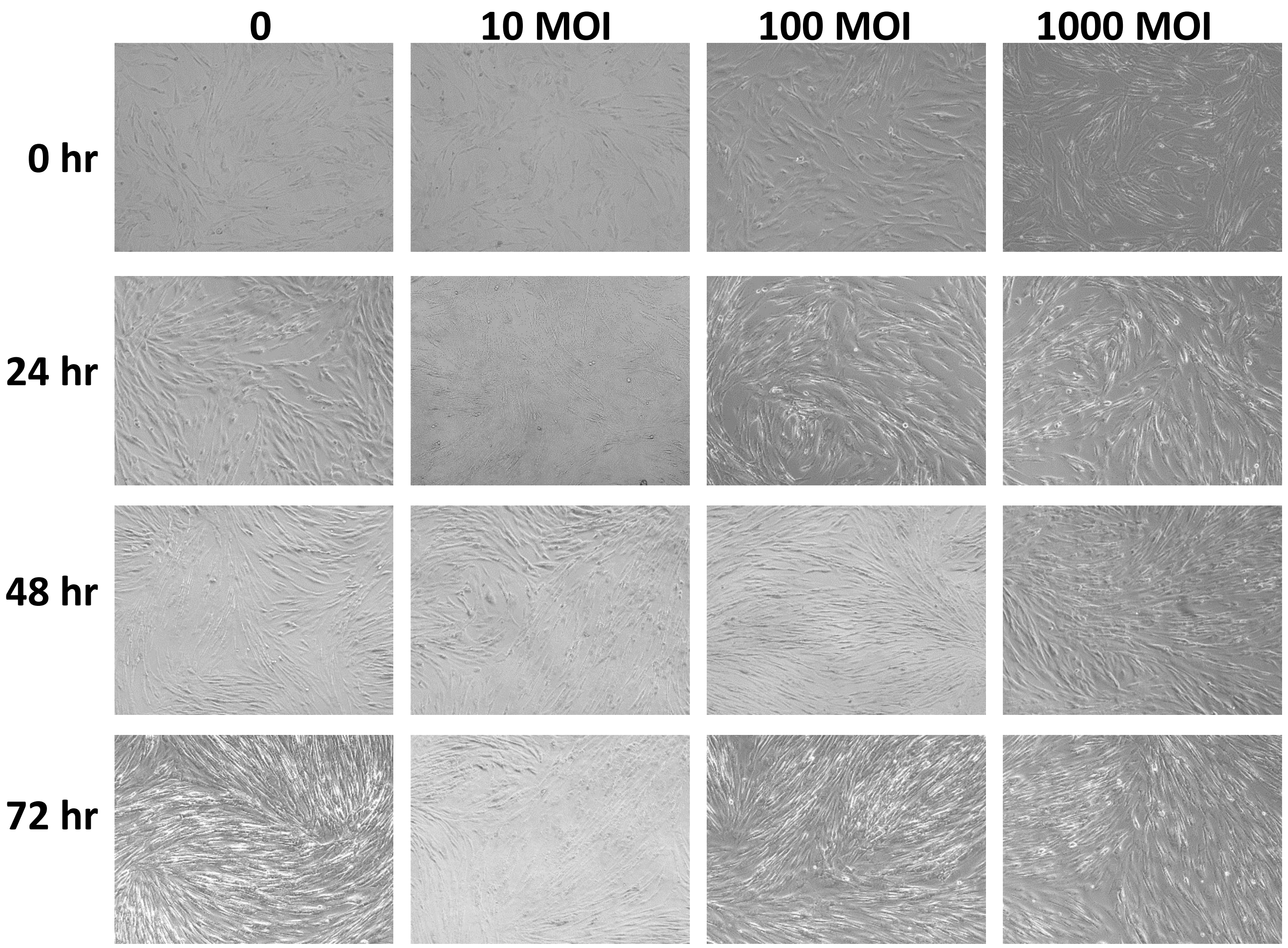

Figure S10: Phase contrast images of CAV2-AU-M2 infections in NCF at different MOIs and time point. NCF cells were infected with CAV-AUM2 at MOI 0, 10,100, and 1000, and the cytopathic effect was visualized at 0 h, 24 h, 48 h and 72 h post-infection by fluorescence microcopy (Keyence) at 10X magnification These is a representation of three independent experiments.

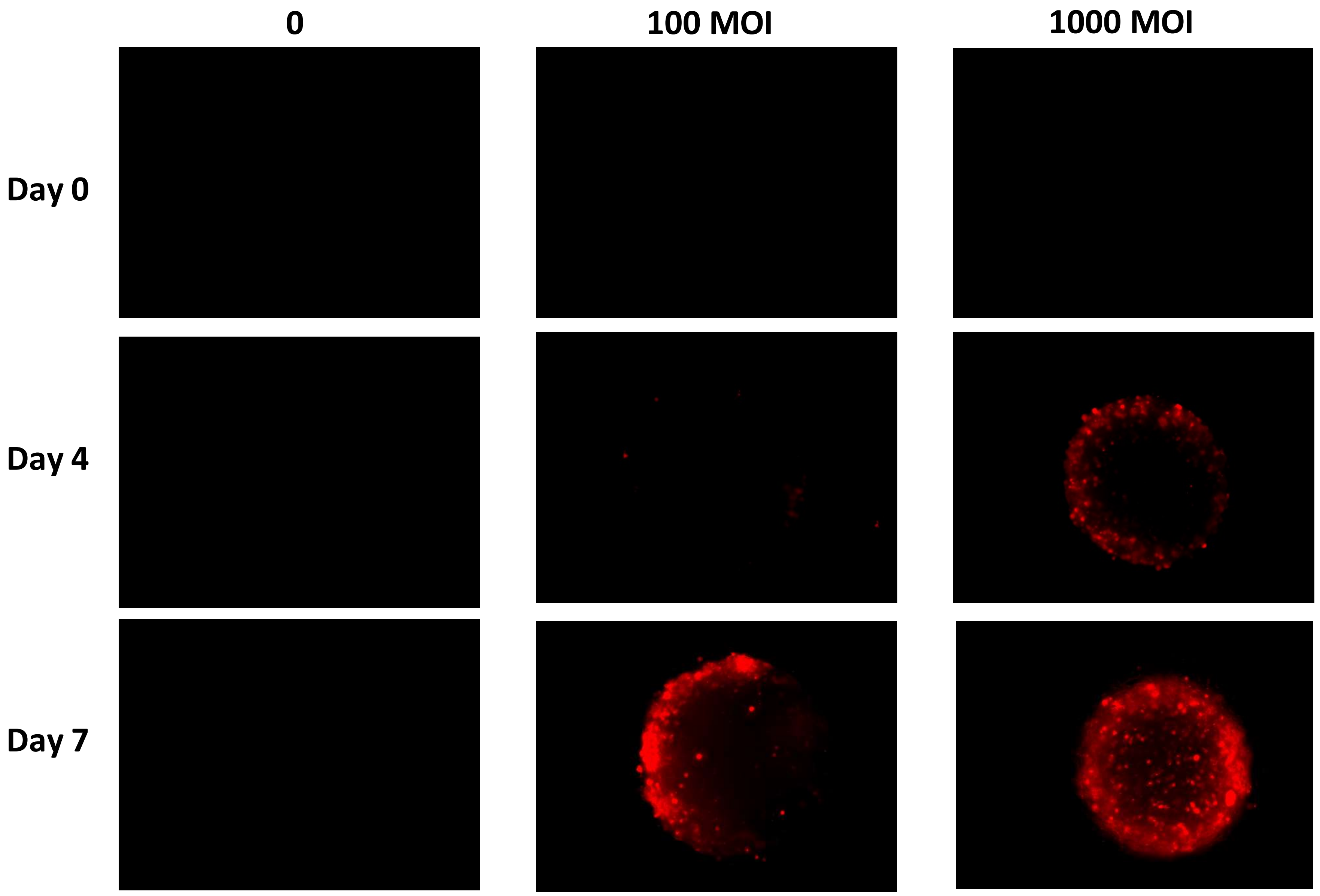

Figure S11: Red fluorescent images of CAV2-AU-M2 infections in D17 cell line spheroids at different MOIs and time point. D17 spheroids were infected with CAV-AUM2 at MOI 0, 100, and 1000, and the dsRed fluorescent signal was visualized at Day 0, 4, and 7 post-infection by fluorescence microcopy (Keyence) at 10X magnification These is a representation of three independent experiments.

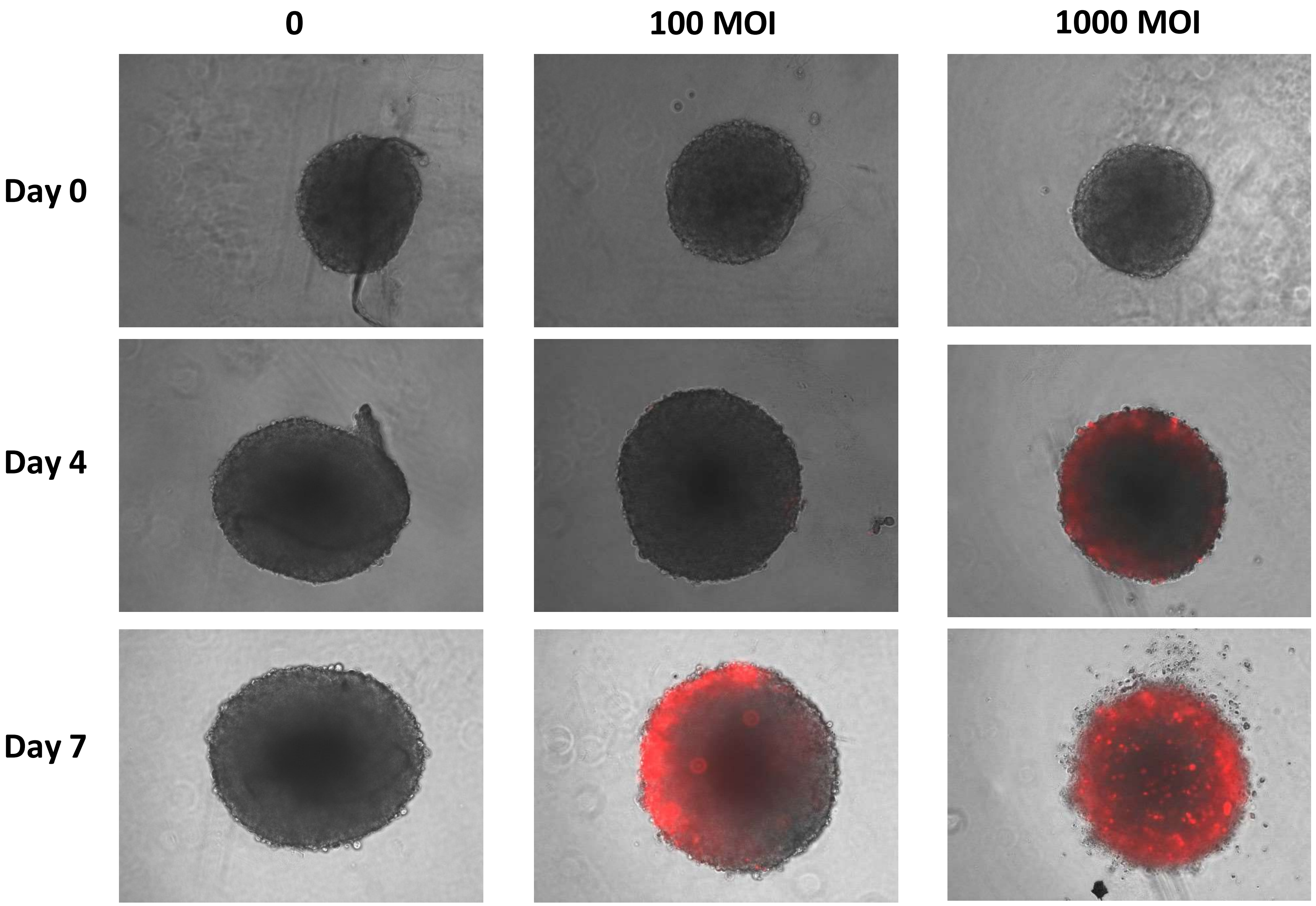

	MOI 0	MOI 100	MOI 1000
Day 0	477	541	550
Day 4	789	713	660
Day 7	829	769	672

Figure S12: Phase contrast images of CAV2-AU-M2 infections in D17 cell line spheroids at different MOIs and time point. D17 spheroids were infected with CAV-AUM2 at MOI 0, 100, and 1000, and the spheroid shrinkage and cytopathic effects were visualized at Day 0, 4, and 7 post-infection by fluorescence microcopy (Keyence) at 10X magnification These is a representation of three independent experiments.

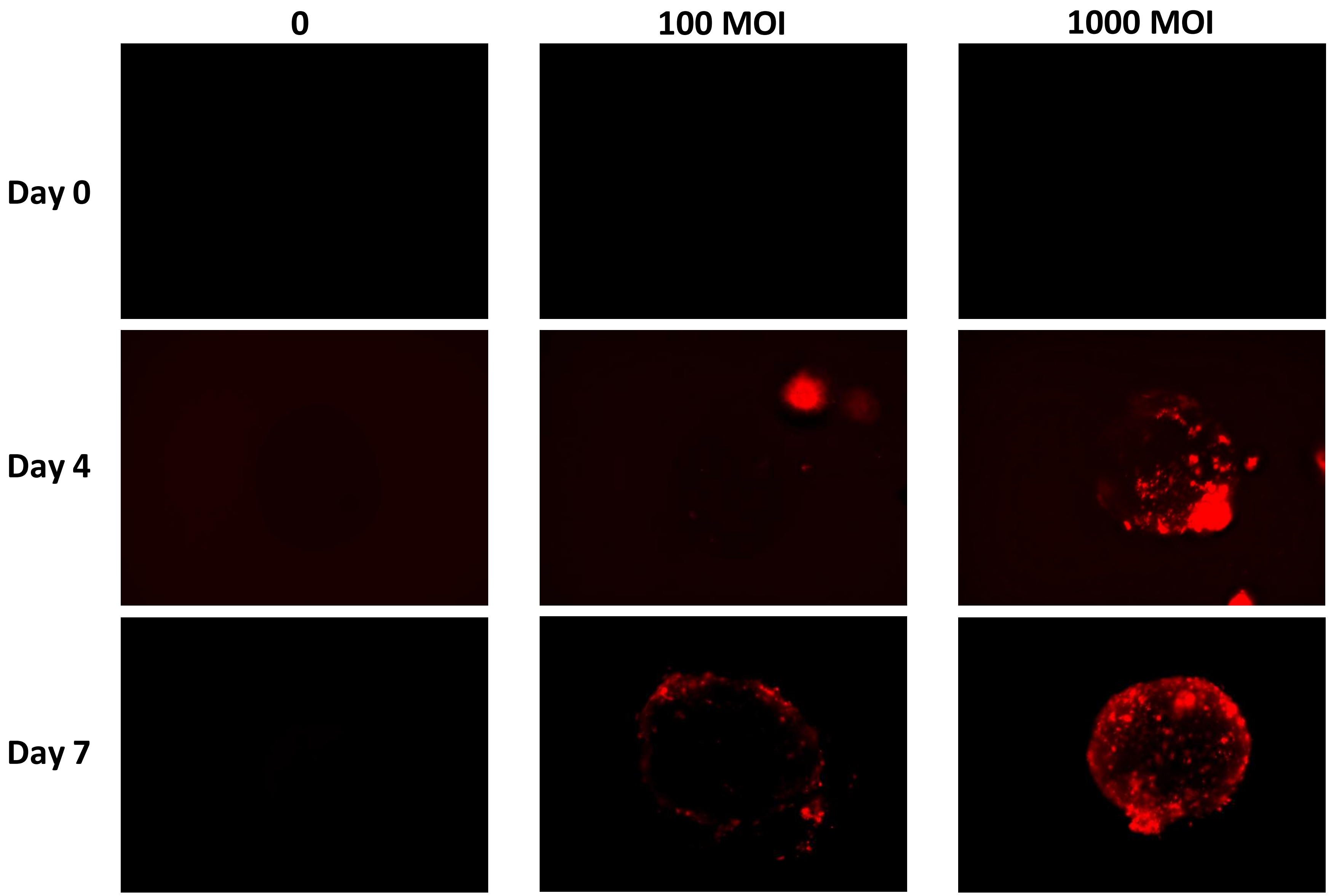

Figure S13: Red fluorescent images of CAV2-AU-M2 infections in CF11 cell line spheroids at different MOIs and time point. CF11 spheroids were infected with CAV-AUM2 at MOI 0, 100, and 1000, and the dsRed fluorescent signal was visualized at Day 0, 4, and 7 post-infection by fluorescence microcopy (Keyence) at 10X magnification These is a representation of three independent experiments.

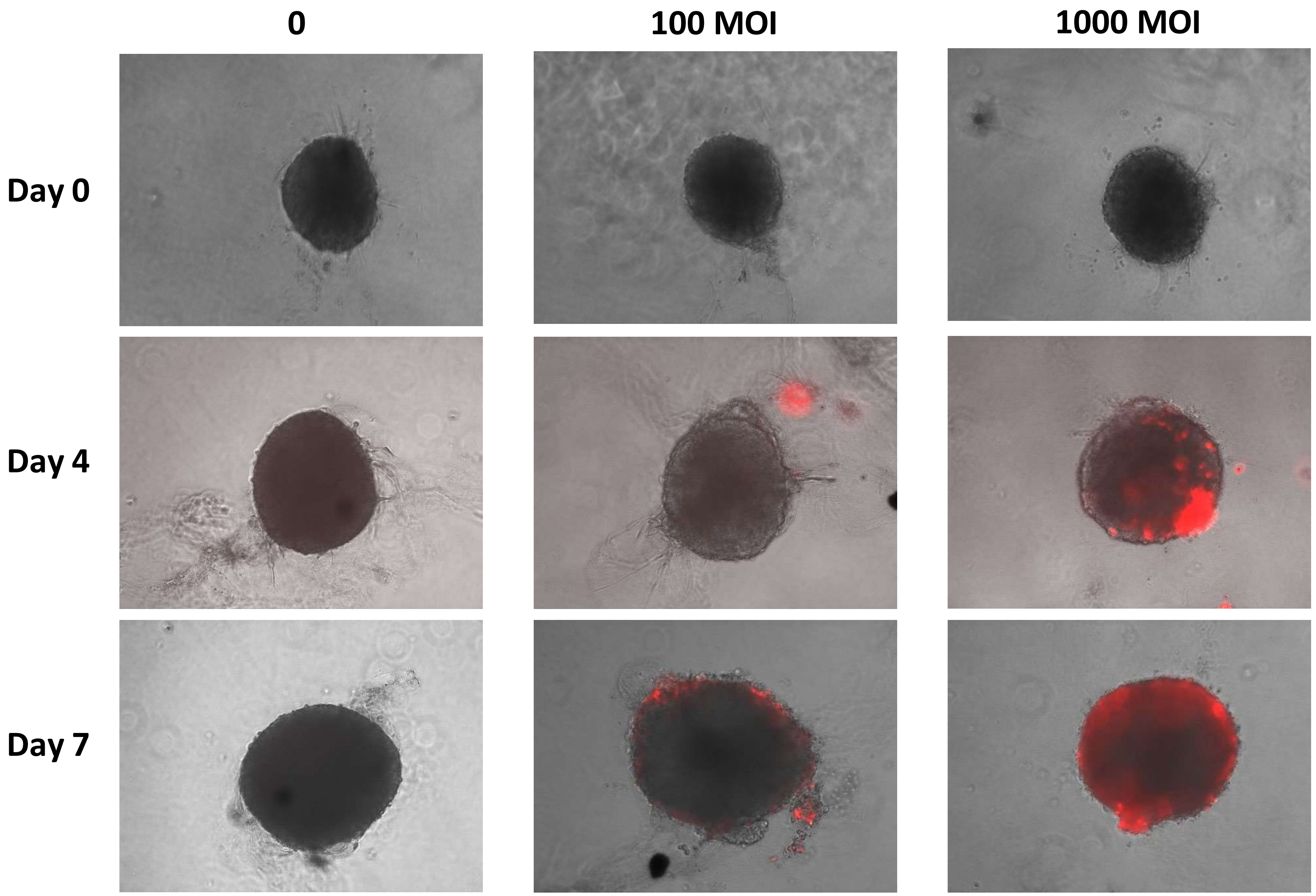

	MOI 0	MOI 100	MOI 1000
Day 0	386	400	408
Day 4	501	507	563
Day 7	644	720	635

Figure S14: Phase contrast images of CAV2-AU-M2 infections in CF11 cell line spheroids at different MOIs and time point. CF11 spheroids were infected with CAV-AUM2 at MOI 0, 100, and 1000, and the spheroid shrinkage and cytopathic effects were visualized at Day 0, 4, and 7 post-infection by fluorescence microcopy (Keyence) at 10X magnification These is a representation of three independent experiments.

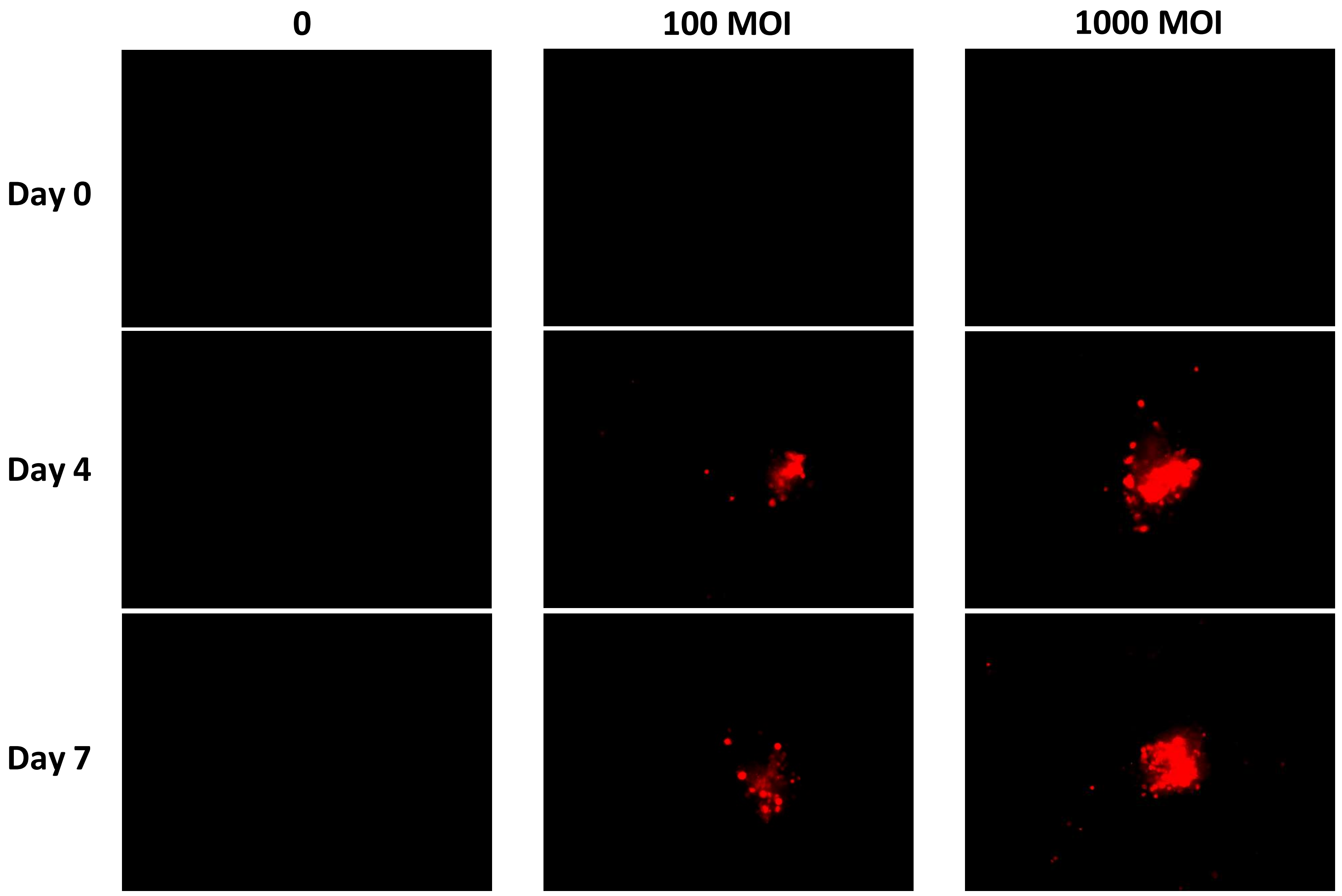

Figure S15: Red fluorescent images of CAV2-AU-M2 infections in D22 cell line spheroids at different MOIs and time point. D22 spheroids were infected with CAV-AUM2 at MOI 0, 100, and 1000, and the dsRed fluorescent signal was visualized at Day 0, 4, and 7 post-infection by fluorescence microcopy (Keyence) at 10X magnification These is a representation of three independent experiments.

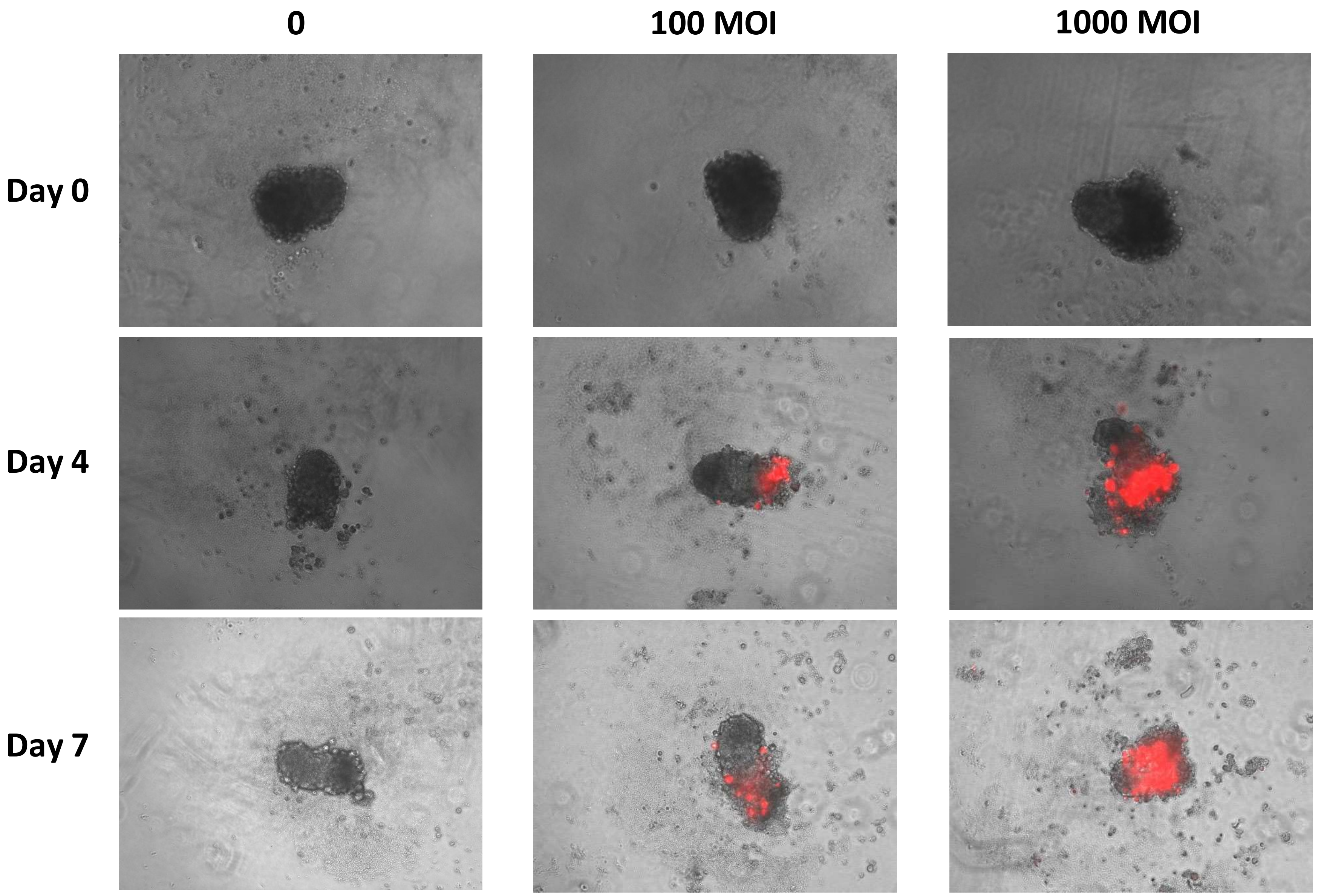

	MOI 0	MOI 100	MOI 1000
Day 0	352	275	397
Day 4	217	372	332
Day 7	329	208	322

Figure S16: Phase contrast images of CAV2-AU-M2 infections in D22 cell line spheroids at different MOIs and time point. D22 spheroids were infected with CAV-AUM2 at MOI 0, 100, and 1000, and the spheroid shrinkage and cytopathic effects were visualized at Day 0, 4, and 7 post-infection by fluorescence microcopy (Keyence) at 10X magnification These is a representation of three independent experiments.

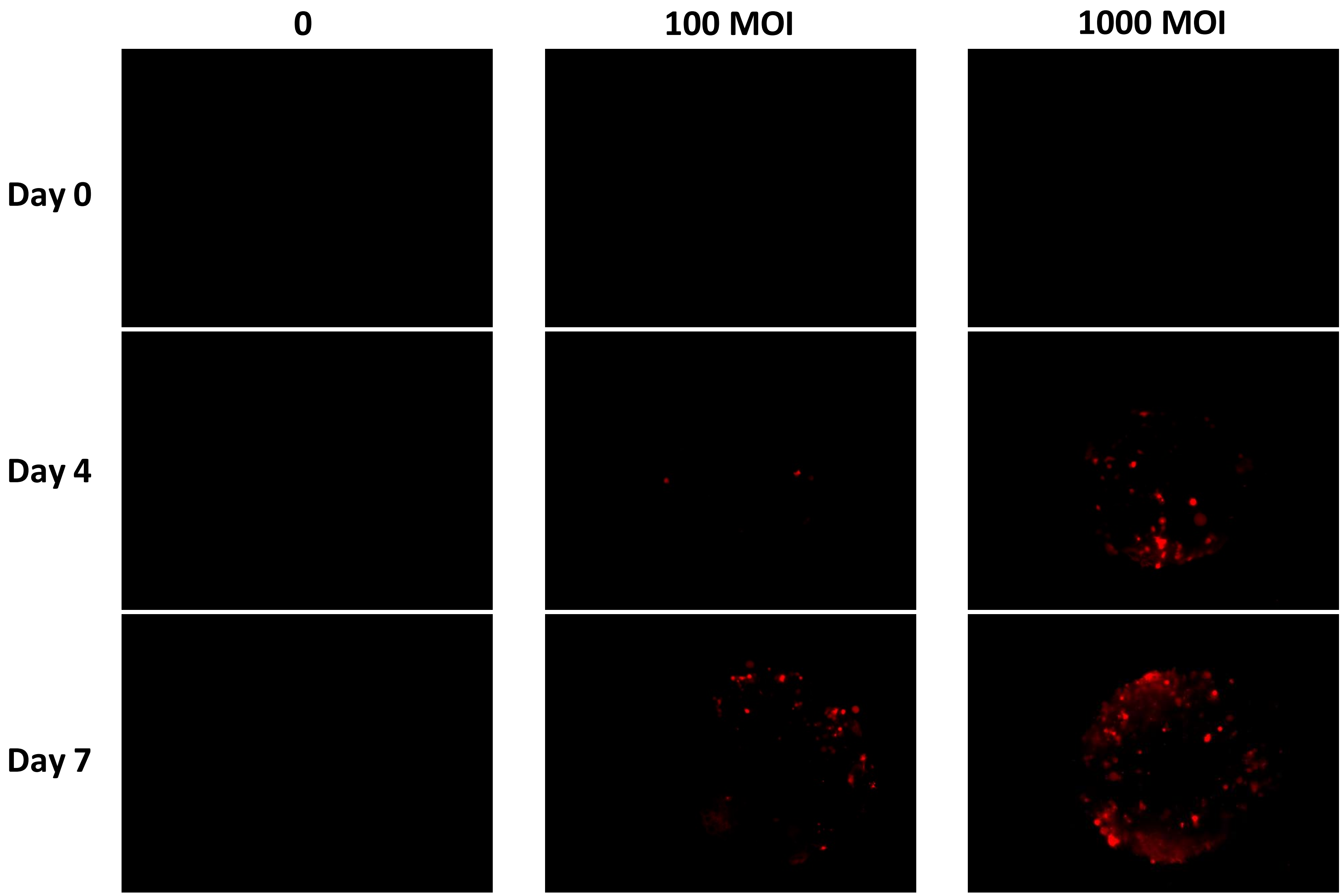

Figure S17: Red fluorescent images of CAV2-AU-M2 infections in MCKOS cell line spheroids at different MOIs and time point. MCKOS spheroids were infected with CAV-AUM2 at MOI 0, 100, and 1000, and the dsRed fluorescent signal was visualized at Day 0, 4, and 7 post-infection by fluorescence microcopy (Keyence) at 10X magnification These is a representation of three independent experiments.

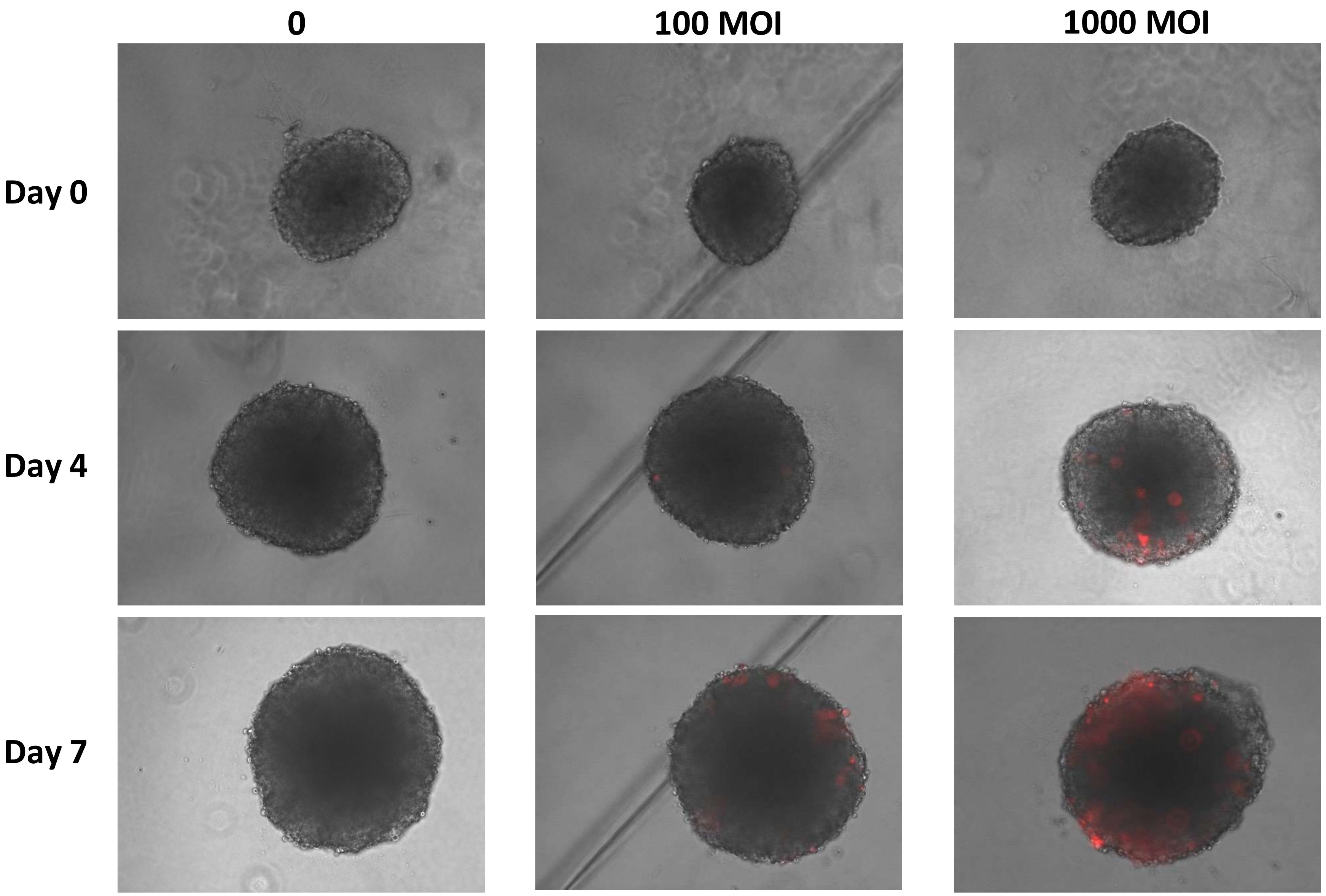

	MOI 0	MOI 100	MOI 1000
Day 0	520	423	505
Day 4	649	662	657
Day 7	739	763	773

Figure S18: Phase contrast images of CAV2-AU-M2 infections in MCKOS cell line spheroids at different MOIs and time point. MCKOS spheroids were infected with CAV-AUM2 at MOI 0, 100, and 1000, and the spheroid shrinkage and cytopathic effects were visualized at Day 0, 4, and 7 post-infection by fluorescence microcopy (Keyence) at 10X magnification These is a representation of three independent experiments.

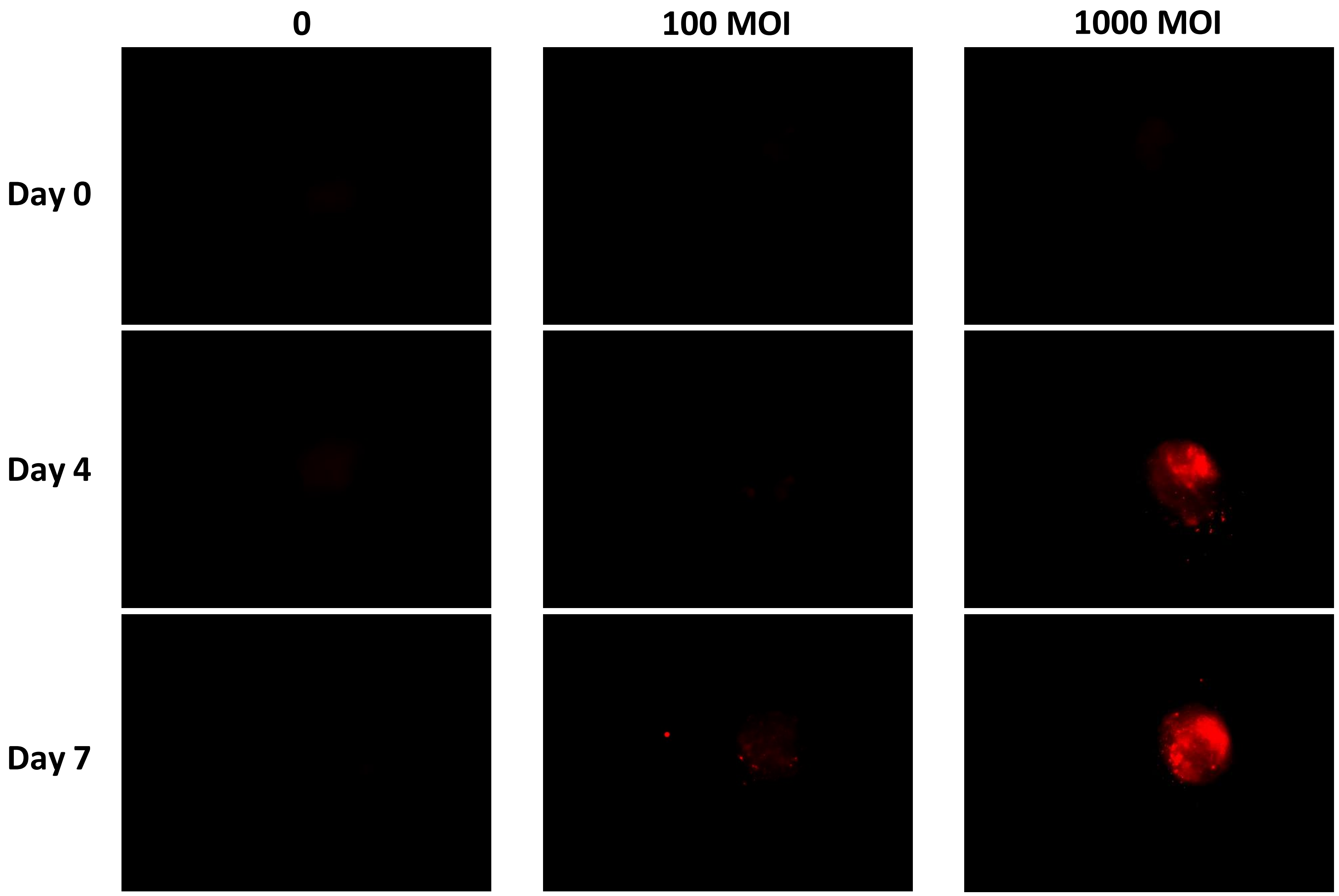

Figure S19: Red fluorescent images of CAV2-AU-M2 infections in NCF spheroids at different MOIs and time point. NCF spheroids were infected with CAV-AUM2 at MOI 0, 100, and 1000, and the dsRed fluorescent signal was visualized at Day 0, 4, and 7 post-infection by fluorescence microcopy (Keyence) at 10X magnification These is a representation of three independent experiments.

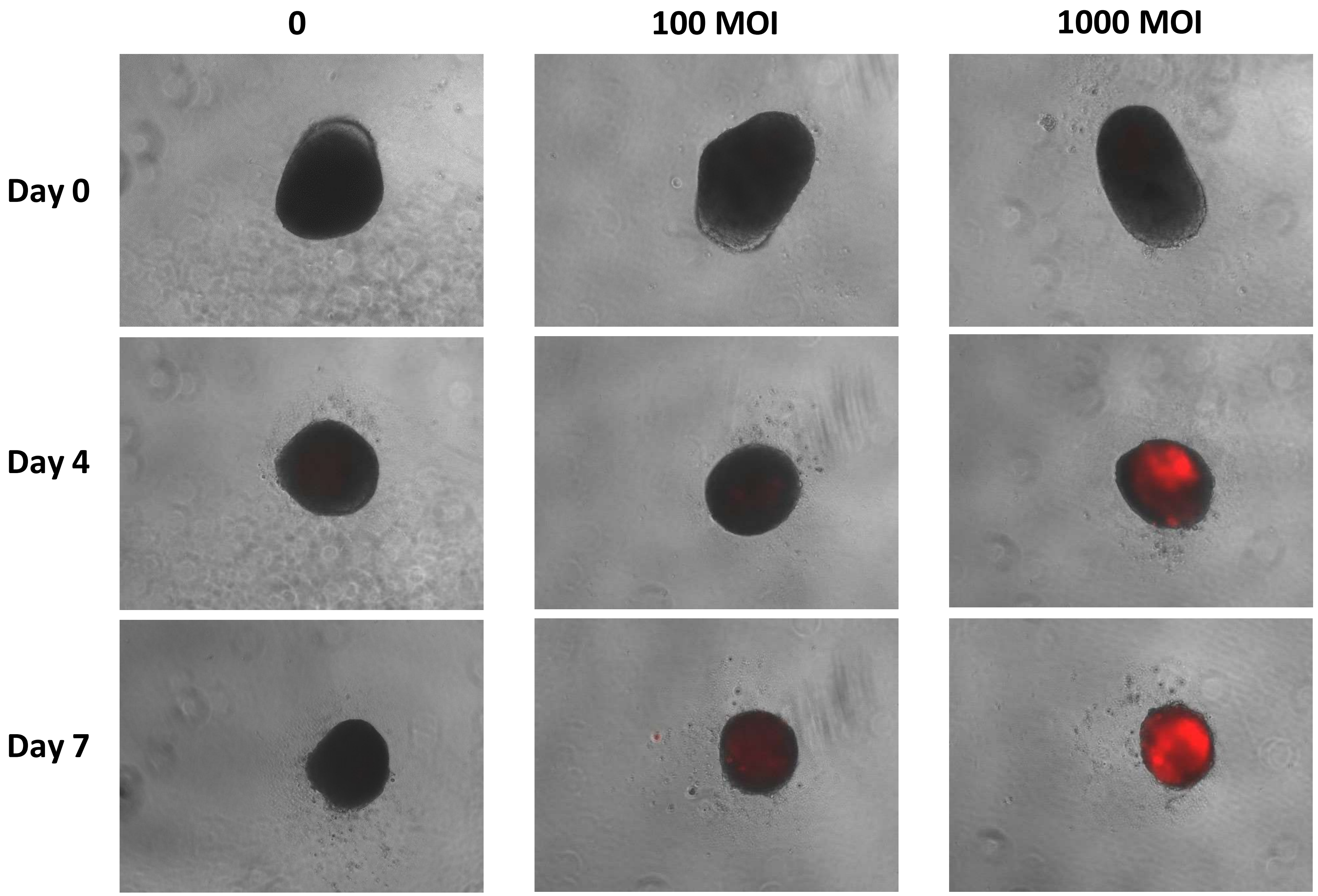

	MOI 0	MOI 100	MOI 1000
Day 0	425	437	394
Day 4	405	382	366
Day 7	339	315	298

Figure S20: Phase contrast images of CAV2-AU-M2 infections in NCF spheroids at different MOIs and time point. NCF spheroids were infected with CAV-AUM2 at MOI 0, 100, and 1000, and the spheroid shrinkage and cytopathic effects were visualized at Day 0, 4, and 7 post-infection by fluorescence microcopy (Keyence) at 10X magnification These is a representation of three independent experiments.

Supplementary Data

Linear Regression for Size and Luminance

We use “y” to represent spheroid size or luminance (2D and 3D) and “$tttme$” to represent hours or days. Moreover, we assume MOI has k levels, i.e. $MOI_1, \ldots, MOI_k$. With this notation, we estimated and tested the following model:

$$y = \beta_0 + \beta_1 MOI_1 + \ldots + \beta_k MOI_k + \beta_{k+1}\, tttme + \beta_{k+2}\, tttme\, \mathbf{11}_{MMMI_1} + \ldots \beta_{2k+2}\, tttme\, \mathbf{11}_{MMMI_k},$$

where the reference level (β_0) is the average y when MOI is zero; $\beta_1, \ldots, \beta_k$ represent the average effect of each level of MOI compared to the reference level β_0; $\mathbf{11}_{MMMI_{ii}}$ is the indicator function which takes the value 1 if in the presence of level MOI_{ii} and 0 otherwise. Therefore, for each cell-line, this model estimates and tests the effect of MOI levels on y as well as the effect of time on y according to different levels of MOI.

We estimate this model for all cell-lines and get the following results:

```
## [1] "Regressions for Spheroid Size condition:"
## [1] "Results for NCF cell-line:"
##
## Call:
## lm(formula = y ~ MOI * Time, data = data)
##
## Residuals:
##     Min      1Q  Median      3Q     Max
## -45.207 -18.725  -2.054  12.081  87.793
##
## Coefficients:
##              Estimate Std. Error t value Pr(>|t|)
## (Intercept)  450.9099    16.7873  26.860  < 2e-16 ***
## MOI100        31.2973    23.7408   1.318 0.201605
## MOI1000      -31.7297    23.7408  -1.337 0.195685
## Time         -14.0360     3.6065  -3.892 0.000841 ***
## MOI100:Time  -10.4144     5.1003  -2.042 0.053921 .
## MOI1000:Time  -0.8919     5.1003  -0.175 0.862858
## ---
## Signif. codes:  0 '***' 0.001 '**' 0.01 '*' 0.05 '.' 0.1 ' ' 1
##
## Residual standard error: 31.02 on 21 degrees of freedom
## Multiple R-squared:  0.8013, Adjusted R-squared:  0.754
## F-statistic: 16.93 on 5 and 21 DF,  p-value: 9.606e-07
##
## [1] "Results for CF11 cell-line:"
##
## Call:
## lm(formula = y ~ MOI * Time, data = data)
##
## Residuals:
```

```
##       Min        1Q    Median        3Q       Max
## -108.892  -20.045    -1.514    27.899    56.279
##
## Coefficients:
##              Estimate Std. Error t value Pr(>|t|)
## (Intercept)   411.405     22.735  18.096 2.75e-14 ***
## MOI100         -9.892     32.152  -0.308    0.761
## MOI1000       -22.653     32.152  -0.705    0.489
## Time           31.829      4.884   6.517 1.86e-06 ***
## MOI100:Time     5.910      6.907   0.856    0.402
## MOI1000:Time    2.905      6.907   0.421    0.678
## ---
## Signif. codes:  0 '***' 0.001 '**' 0.01 '*' 0.05 '.' 0.1 ' ' 1
##
## Residual standard error: 42.02 on 21 degrees of freedom
## Multiple R-squared: 0.8801, Adjusted R-squared:  0.8516
## F-statistic: 30.84 on 5 and 21 DF,  p-value: 5.398e-09
##
## [1] "Results for D17 cell-line:"
##
## Call:
## lm(formula = y ~ MOI * Time, data = data)
##
## Residuals:
##    Min     1Q Median     3Q    Max
## -64.36 -28.60  -6.00  32.26  83.50
##
## Coefficients:
##              Estimate Std. Error t value Pr(>|t|)
## (Intercept)   532.833     23.703  22.480 3.58e-16 ***
## MOI100         25.698     33.521   0.767  0.45184
## MOI1000        47.604     33.521   1.420  0.17025
## Time           43.167      5.092   8.477 3.21e-08 ***
## MOI100:Time   -14.554      7.202  -2.021  0.05622 .
## MOI1000:Time  -20.892      7.202  -2.901  0.00854 **
## ---
## Signif. codes:  0 '***' 0.001 '**' 0.01 '*' 0.05 '.' 0.1 ' ' 1
##
## Residual standard error: 43.81 on 21 degrees of freedom
## Multiple R-squared: 0.8562, Adjusted R-squared:  0.822
## F-statistic: 25.02 on 5 and 21 DF,  p-value: 3.505e-08
##
## [1] "Results for D22 cell-line:"
##
## Call:
## lm(formula = y ~ MOI * Time, data = data)
##
## Residuals:
##       Min        1Q    Median        3Q       Max
## -100.577   -26.243     4.604    40.797    75.234
```

```
##
## Coefficients:
##              Estimate Std. Error t value Pr(>|t|)
## (Intercept)   329.455     28.603  11.518 1.54e-10 ***
## MOI100         46.122     40.451   1.140    0.267
## MOI1000        18.842     40.451   0.466    0.646
## Time          -10.518      6.145  -1.712    0.102
## MOI100:Time    -9.185      8.690  -1.057    0.303
## MOI1000:Time    6.104      8.690   0.702    0.490
## ---
## Signif. codes:  0 '***' 0.001 '**' 0.01 '*' 0.05 '.' 0.1  ' ' 1
##
## Residual standard error: 52.86 on 21 degrees of freedom
## Multiple R-squared: 0.4416, Adjusted R-squared:  0.3086
## F-statistic: 3.321 on 5 and 21 DF,  p-value: 0.02297
##
## [1] "Results for MCKOS cell-line:"
##
## Call:
## lm(formula = y ~ MOI * Time, data = data)
##
## Residuals:
##     Min      1Q  Median      3Q     Max
## -164.04  -33.75  16.19   61.49   90.26
##
## Coefficients:
##              Estimate Std. Error t value Pr(>|t|)
## (Intercept)    537.81      46.92  11.463 1.69e-10 ***
## MOI100         -69.19      66.35  -1.043    0.309
## MOI1000        -44.92      66.35  -0.677    0.506
## Time            16.69      10.08   1.656    0.113
## MOI100:Time     15.66      14.25   1.098    0.284
## MOI1000:Time    10.43      14.25   0.732    0.472
## ---
## Signif. codes: 0 '***' 0.001 '**' 0.01 '*' 0.05 '.' 0.1  ' ' 1
##
## Residual standard error: 86.71 on 21 degrees of freedom
## Multiple R-squared: 0.4923, Adjusted R-squared:  0.3714
## F-statistic: 4.073 on 5 and 21 DF,  p-value: 0.009665
##
## [1] "Regressions for LDH Cytotoxicity Data 2D condition:"
## [1] "Results for NCF cell-line:"
##
## Call:
## lm(formula = y ~ MOI * Time, data = data)
##
## Residuals:
##     Min      1Q  Median      3Q     Max
## -4349.0 -1390.1  -219.5  1521.6  5333.9
##
```

```
## Coefficients:
##               Estimate Std. Error t value Pr(>|t|)
## (Intercept)    8485.10    1223.85   6.933 2.32e-08 ***
## MOI10         -1925.40    1730.79  -1.112   0.2726
## MOI100         1798.26    1730.79   1.039   0.3051
## MOI1000       -1312.83    1730.79  -0.759   0.4526
## Time             30.06      27.26   1.103   0.2767
## MOI10:Time       79.56      38.55   2.064   0.0456 *
## MOI100:Time     -33.74      38.55  -0.875   0.3866
## MOI1000:Time     65.41      38.55   1.697   0.0975 .
## ---
## Signif. codes:  0 '***' 0.001 '**' 0.01 '*' 0.05 '.' 0.1  ' ' 1
##
## Residual standard error: 2534 on 40 degrees of freedom
## Multiple R-squared: 0.436,  Adjusted R-squared:  0.3372
## F-statistic: 4.417 on 7 and 40 DF,  p-value: 0.001033
##
## [1] "Results for CF11 cell-line:"
##
## Call:
## lm(formula = y ~ MOI * Time, data = data)
##
## Residuals:
##      Min       1Q   Median       3Q      Max
## -17076.8  -3550.9    307.9   2065.7  22265.3
##
## Coefficients:
##               Estimate Std. Error t value Pr(>|t|)
## (Intercept)   9238.533   3116.564   2.964  0.00509 **
## MOI10            2.267   4407.487   0.001  0.99959
## MOI100       -3185.767   4407.487  -0.723  0.47400
## MOI1000      -7189.367   4407.487  -1.631  0.11070
## Time            32.862     69.411   0.473  0.63847
## MOI10:Time     -36.044     98.163  -0.367  0.71541
## MOI100:Time     75.815     98.163   0.772  0.44445
## MOI1000:Time   446.256     98.163   4.546 4.97e-05 ***
## ---
## Signif. codes:  0 '***' 0.001 '**' 0.01 '*' 0.05 '.' 0.1  ' ' 1
##
## Residual standard error: 6452 on 40 degrees of freedom
## Multiple R-squared:  0.6361, Adjusted R-squared:  0.5724
## F-statistic: 9.989 on 7 and 40 DF,  p-value: 3.8e-07
##
## [1] "Results for D17 cell-line:"
##
## Call:
## lm(formula = y ~ MOI * Time, data = data)
##
## Residuals:
##    Min      1Q Median      3Q     Max
```

```
## -22988  -2873   -581   3088  33288
##
## Coefficients:
##               Estimate Std. Error t value Pr(>|t|)
## (Intercept)   12238.83    4821.11   2.539   0.0151 *
## MOI10          2057.87    6818.08   0.302   0.7643
## MOI100        -2040.63    6818.08  -0.299   0.7663
## MOI1000      -16175.00    6818.08  -2.372   0.0226 *
## Time            -60.31     107.37  -0.562   0.5775
## MOI10:Time       15.06     151.85   0.099   0.9215
## MOI100:Time     120.55     151.85   0.794   0.4320
## MOI1000:Time   1059.22     151.85   6.975 2.03e-08 ***
## ---
## Signif. codes:  0 '***' 0.001 '**' 0.01 '*' 0.05 '.' 0.1 ' ' 1
##
## Residual standard error: 9981 on 40 degrees of freedom
## Multiple R-squared: 0.7578, Adjusted R-squared:  0.7154
## F-statistic: 17.88 on 7 and 40 DF,  p-value: 1.66e-10
##
## [1] "Results for D22 cell-line:"
##
## Call:
## lm(formula = y ~ MOI * Time, data = data)
##
## Residuals:
##    Min     1Q Median     3Q    Max
## -20766  -6944   1327   6617  32336
##
## Coefficients:
##              Estimate  Std. Error t value Pr(>|t|)
## (Intercept)   5861.47    5228.50   1.121    0.269
## MOI10        -2426.80    7394.21  -0.328    0.744
## MOI100       -2458.23    7394.21  -0.332    0.741
## MOI1000        124.70    7394.21   0.017    0.987
## Time           918.07     116.45   7.884 1.14e-09 ***
## MOI10:Time      58.72     164.68   0.357    0.723
## MOI100:Time     72.07     164.68   0.438    0.664
## MOI1000:Time   -57.85     164.68  -0.351    0.727
## ---
## Signif. codes:  0 '***' 0.001 '**' 0.01 '*' 0.05 '.' 0.1  ' ' 1
##
## Residual standard error: 10820 on 40 degrees of freedom
## Multiple R-squared:  0.8665, Adjusted R-squared:  0.8432
## F-statistic:  37.1 on 7 and 40 DF,  p-value: 1.521e-15
##
## [1] "Results for MCKOS cell-line:"
##
## Call:
## lm(formula = y ~ MOI * Time, data = data)
##
```

```
## Residuals:
##     Min      1Q  Median      3Q     Max
## -8014.5 -3498.7   300.2  1951.6  8232.6
##
## Coefficients:
##               Estimate Std. Error t value Pr(>|t|)
## (Intercept)  10791.567   1865.895   5.784 9.52e-07 ***
## MOI10        -1476.867   2638.774  -0.560    0.579
## MOI100        2276.333   2638.774   0.863    0.393
## MOI1000        595.333   2638.774   0.226    0.823
## Time            53.274     41.557   1.282    0.207
## MOI10:Time       3.554     58.770   0.060    0.952
## MOI100:Time    -51.458     58.770  -0.876    0.386
## MOI1000:Time    44.083     58.770   0.750    0.458
## ---
## Signif. codes:  0 '***' 0.001 '**' 0.01 '*' 0.05 '.' 0.1 ' ' 1
##
## Residual standard error: 3863 on 40 degrees of freedom
## Multiple R-squared:  0.261,  Adjusted R-squared:  0.1316
## F-statistic: 2.018 on 7 and 40 DF,  p-value: 0.07663
##
## [1] "Regressions for LDH Cytotoxicity Data 3D condition:"
## [1] "Results for NCF cell-line:"
##
## Call:
## lm(formula = y ~ MOI * Time, data = data)
##
## Residuals:
##     Min      1Q  Median      3Q     Max
## -1801.4 -1192.0   -48.9   703.2  3878.9
##
## Coefficients:
##              Estimate Std. Error t value Pr(>|t|)
## (Intercept)   9798.91     796.04  12.310 4.55e-11 ***
## MOI100       -1192.67    1125.77  -1.059    0.301
## MOI1000      -1879.48    1125.77  -1.670    0.110
## Time           238.66     171.02   1.396    0.177
## MOI100:Time    -59.75     241.85  -0.247    0.807
## MOI1000:Time    87.59     241.85   0.362    0.721
## ---
## Signif. codes:  0 '***' 0.001 '**' 0.01 '*' 0.05 '.' 0.1 ' ' 1
##
## Residual standard error: 1471 on 21 degrees of freedom
## Multiple R-squared:  0.3794, Adjusted R-squared:  0.2317
## F-statistic: 2.568 on 5 and 21 DF,  p-value: 0.05792
##
## [1] "Results for CF11 cell-line:"
##
## Call:
## lm(formula = y ~ MOI * Time, data = data)
```

```
##
## Residuals:
##     Min      1Q  Median      3Q     Max
## -3043.3 -1424.5   406.1  1186.1  3968.7
##
## Coefficients:
##              Estimate Std. Error t value Pr(>|t|)
## (Intercept)   1520.07    1132.07   1.343    0.194
## MOI100        -301.13    1600.98  -0.188    0.853
## MOI1000       -613.68    1600.98  -0.383    0.705
## Time           644.36     243.21   2.649    0.015 *
## MOI100:Time    -53.95     343.95  -0.157    0.877
## MOI1000:Time   386.63     343.95   1.124    0.274
## ---
## Signif. codes:  0 '***' 0.001 '**' 0.01 '*' 0.05 '.' 0.1 ' ' 1
##
## Residual standard error: 2092 on 21 degrees of freedom
## Multiple R-squared: 0.6086, Adjusted R-squared:  0.5155
## F-statistic: 6.532 on 5 and 21 DF,  p-value: 0.0008232
##
## [1] "Results for D17 cell-line:"
##
## Call:
## lm(formula = y ~ MOI * Time, data = data)
##
## Residuals:
##     Min     1Q  Median     3Q     Max
## -1931.5  -930.5  -561.2  696.1  2864.5
##
## Coefficients:
##              Estimate Std. Error t value Pr(>|t|)
## (Intercept)   3933.15     750.85   5.238 3.42e-05 ***
## MOI100       -1349.91    1061.86  -1.271    0.218
## MOI1000      -1046.60    1061.86  -0.986    0.336
## Time            48.84     161.31   0.303    0.765
## MOI100:Time    167.42     228.12   0.734    0.471
## MOI1000:Time   256.69     228.12   1.125    0.273
## ---
## Signif. codes: 0 '***' 0.001 '**' 0.01 '*' 0.05 '.' 0.1 ' ' 1
##
## Residual standard error: 1388 on 21 degrees of freedom
## Multiple R-squared:  0.2489, Adjusted R-squared:  0.07003
## F-statistic: 1.392 on 5 and 21 DF, p-value: 0.2677
##
## [1] "Results for D22 cell-line:"
##
## Call:
## lm(formula = y ~ MOI * Time, data = data)
##
## Residuals:
```

```
##      Min       1Q   Median       3Q      Max
## -3473.5   -655.6    210.6    938.5   2669.1
##
## Coefficients:
##              Estimate Std. Error t value Pr(>|t|)
## (Intercept)    8540.9      946.9   9.020 1.14e-08 ***
## MOI100        -1976.7     1339.1  -1.476   0.1548
## MOI1000        1010.5     1339.1   0.755   0.4588
## Time            447.2      203.4   2.198   0.0393 *
## MOI100:Time     319.4      287.7   1.110   0.2795
## MOI1000:Time   -159.0      287.7  -0.553   0.5862
## ---
## Signif. codes:  0 '***' 0.001 '**' 0.01 '*' 0.05 '.' 0.1 ' ' 1
##
## Residual standard error: 1750 on 21 degrees of freedom
## Multiple R-squared: 0.5264, Adjusted R-squared:  0.4136
## F-statistic: 4.668 on 5 and 21 DF,  p-value: 0.005066
##
## [1] "Results for MCKOS cell-line:"
##
## Call:
## lm(formula = y ~ MOI * Time, data = data)
##
## Residuals:
##     Min      1Q  Median      3Q     Max
## -2286.2  -936.6  -398.7   891.2  2509.8
##
## Coefficients:
##              Estimate Std. Error t value Pr(>|t|)
## (Intercept)    5030.8      822.7   6.115 4.56e-06 ***
## MOI100        -2447.6     1163.5  -2.104   0.0476 *
## MOI1000       -2144.3     1163.5  -1.843   0.0795 .
## Time           -136.9      176.7  -0.775   0.4472
## MOI100:Time     353.2      250.0   1.413   0.1723
## MOI1000:Time    442.5      250.0   1.770   0.0912 .
## ---
## Signif. codes: 0 '***' 0.001 '**' 0.01 '*' 0.05 '.' 0.1 ' ' 1
##
## Residual standard error: 1520 on 21 degrees of freedom
## Multiple R-squared: 0.2678, Adjusted R-squared:  0.09342
## F-statistic: 1.536 on 5 and 21 DF,  p-value: 0.2215
```

Multiple Proportion Tests for Binding and Inhibition Assays

To test average percentages (proportions), let p_2 represent the percentage for which we want to understand if it is greater than another percentage that we denote as p_1. For example, we are interested in testing if the percentage for "D17 AUM2 super" (p_2) is larger than the average percentage (proportion) for "D17 AUM1 super" (p_1). Then we would want to test the set of hypotheses:

Comparing each test to the Bonferroni corrected significance level $\alpha_{b\_2} = 0.05/8$ and knowing that the sample size on which each percentage is computed is $n = 10{,}000$, we obtain the following results:

```
## [1] "PD-1 Binding Assay Media"

## [1] "D17 AUM2 super vs D17 NI super"

##
## 2-sample test for equality of proportions with continuity correction
##
## data:  c(9253, 12) out of c(10000, 10000)
## X-squared = 17168, df = 1, p-value < 2.2e-16
## alternative hypothesis: greater
## 95 percent confidence interval:
##  0.9196382 1.0000000
## sample estimates:
## prop 1 prop 2
## 0.9253 0.0012

## [1] "D17 AUM2 super vs D17 AUM1 super"

##
## 2-sample test for equality of proportions with continuity correction
##
## data:  c(9253, 9) out of c(10000, 10000)
## X-squared = 17180, df = 1, p-value < 2.2e-16
## alternative hypothesis: greater
## 95 percent confidence interval:
##  0.9199475 1.0000000
## sample estimates:
## prop 1 prop 2
## 0.9253 0.0009

## [1] "CF11 AUM2 super vs CF11 NI super"

##
## 2-sample test for equality of proportions with continuity correction
##
## data:  c(51, 17) out of c(10000, 10000)
## X-squared = 16.069, df = 1, p-value = 3.053e-05
## alternative hypothesis: greater
## 95 percent confidence interval:
##  0.001946504 1.000000000
## sample estimates:
## prop 1 prop 2
## 0.0051 0.0017

## [1] "CF11 AUM2 super vs CF11 AUM1 super"
```

```
##
## 2-sample test for equality of proportions with continuity correction
##
## data:  c(51, 9) out of c(10000, 10000)
## X-squared = 28.101, df = 1, p-value = 5.757e-08
## alternative hypothesis: greater
## 95 percent confidence interval:
##  0.002828753 1.000000000
## sample estimates:
## prop 1 prop 2
## 0.0051 0.0009

## [1] "MC-KOS AUM2 super vs MC-KOS NI super"

##
## 2-sample test for equality of proportions with continuity correction
##
## data:  c(55, 66) out of c(10000, 10000)
## X-squared = 0.83148, df = 1, p-value = 0.8191
## alternative hypothesis: greater
## 95 percent confidence interval:
##  -0.003003812  1.000000000
## sample estimates:
## prop 1 prop 2
## 0.0055 0.0066

## [1] "MC-KOS AUM2 super vs MC-KOS AUM1 super"

##
## 2-sample test for equality of proportions with continuity correction
##
## data: c(55, 56) out of c(10000, 10000)
## X-squared = 0, df = 1, p-value = 0.5
## alternative hypothesis: greater
## 95 percent confidence interval:
##  -0.001928145  1.000000000
## sample estimates:
## prop 1 prop 2
## 0.0055 0.0056

## [1] "D22 AUM2 super vs D22 NI super"

##
## 2-sample test for equality of proportions with continuity correction
##
## data:  c(36, 60) out of c(10000, 10000)
## X-squared = 5.537, df = 1, p-value = 0.9907
## alternative hypothesis: greater
## 95 percent confidence interval:
##  -0.004107506  1.000000000
## sample estimates:
```

```
## prop 1 prop 2
## 0.0036 0.0060
```

```
## [1] "D22 AUM2 super vs D22 AUM1 super"
```

```
##
## 2-sample test for equality of proportions with continuity correction
##
## data:  c(36, 64) out of c(10000, 10000)
## X-squared = 7.3266, df = 1, p-value = 0.9966
## alternative hypothesis: greater
## 95 percent confidence interval:
##  -0.004540413  1.000000000
## sample estimates:
## prop 1 prop 2
## 0.0036 0.0064
```

```
## [1] "PD-1 Binding Assay CL"
```

```
## [1] "D17 AUM2 CL vs D17 NI CL"
```

```
##
## 2-sample test for equality of proportions with continuity correction
##
## data:  c(6448, 10) out of c(10000, 10000)
## X-squared = 9475.8, df = 1, p-value < 2.2e-16
## alternative hypothesis: greater
## 95 percent confidence interval:
##  0.635811 1.000000
## sample estimates:
## prop 1 prop 2
## 0.6448 0.0010
```

```
## [1] "D17 AUM2 CL vs D17 AUM1 CL"
```

```
##
## 2-sample test for equality of proportions with continuity correction
##
## data:  c(6448, 6) out of c(10000, 10000)
## X-squared = 9490.7, df = 1, p-value < 2.2e-16
## alternative hypothesis: greater
## 95 percent confidence interval:
##  0.6362179 1.0000000
## sample estimates:
## prop 1 prop 2
## 0.6448 0.0006
```

```
## [1] "CF11 AUM2 CL vs CF11 NI CL"
```

```
##
## 2-sample test for equality of proportions with continuity correction
##
```

```
## data:  c(1195, 9) out of c(10000, 10000)
## X-squared = 1241, df = 1, p-value < 2.2e-16
## alternative hypothesis: greater
## 95 percent confidence interval:
##  0.1131417 1.0000000
## sample estimates:
## prop 1 prop 2
## 0.1195 0.0009

## [1] "CF11 AUM2 CL vs CF11 AUM1 CL"

##
## 2-sample test for equality of proportions with continuity correction
##
## data:  c(1195, 11) out of c(10000, 10000)
## X-squared = 1234.9, df = 1, p-value < 2.2e-16
## alternative hypothesis: greater
## 95 percent confidence interval:
##  0.1129367 1.0000000
## sample estimates:
## prop 1 prop 2
## 0.1195 0.0011

## [1] "MC-KOS AUM2 CL vs MC-KOS NI CL"

##
## 2-sample test for equality of proportions with continuity correction
##
## data:  c(1290, 68) out of c(10000, 10000)
## X-squared = 1177.8, df = 1, p-value < 2.2e-16
## alternative hypothesis: greater
## 95 percent confidence interval:
##  0.1164232 1.0000000
## sample estimates:
## prop 1 prop 2
## 0.1290 0.0068

## [1] "MC-KOS AUM2 CL vs MC-KOS AUM1 CL"

##
## 2-sample test for equality of proportions with continuity correction
##
## data:  c(1290, 51) out of c(10000, 10000)
## X-squared = 1225.1, df = 1, p-value < 2.2e-16
## alternative hypothesis: greater
## 95 percent confidence interval:
##  0.1181633 1.0000000
## sample estimates:
## prop 1 prop 2
## 0.1290 0.0051
```

```
## [1] "D22 AUM2 CL vs D22 NI CL"

##
## 2-sample test for equality of proportions with continuity correction
##
## data:  c(5295, 14) out of c(10000, 10000)
## X-squared = 7148.8, df = 1, p-value < 2.2e-16
## alternative hypothesis: greater
## 95 percent confidence interval:
##  0.5197671 1.0000000
## sample estimates:
## prop 1 prop 2
## 0.5295 0.0014

## [1] "D22 AUM2 CL vs D22 AUM1 CL"

##
## 2-sample test for equality of proportions with continuity correction
##
## data:  c(5295, 63) out of c(10000, 10000)
## X-squared = 6975.8, df = 1, p-value < 2.2e-16
## alternative hypothesis: greater
## 95 percent confidence interval:
##  0.5147875 1.0000000
## sample estimates:
## prop 1 prop 2
## 0.5295 0.0063

## [1] "PD-L1 Inhibition Assay"

## [1] "D17 AUM2 super vs secondary Ab PDL1"

##
## 2-sample test for equality of proportions with continuity correction
##
## data:  c(7104, 7610) out of c(10000, 10000)
## X-squared = 65.577, df = 1, p-value = 2.794e-16
## alternative hypothesis: less
## 95 percent confidence interval:
##  -1.0000000 -0.0402594
## sample estimates:
## prop 1 prop 2
## 0.7104 0.7610

## [1] "D17 AUM2 CL vs secondary Ab PDL1"

##
## 2-sample test for equality of proportions with continuity correction
##
## data:  c(7266, 7610) out of c(10000, 10000)
## X-squared = 30.869, df = 1, p-value = 1.38e-08
## alternative hypothesis: less
```

```
## 95 percent confidence interval:
##  -1.00000000 -0.02415335
## sample estimates:
## prop 1 prop 2
## 0.7266 0.7610

## [1] "CF11 AUM2 super vs secondary Ab PDL1"

##
## 2-sample test for equality of proportions with continuity correction
##
## data:  c(7635, 7610) out of c(10000, 10000)
## X-squared = 0.15892, df = 1, p-value = 0.6549
## alternative hypothesis: less
## 95 percent confidence interval:
##  -1.0000000  0.0125026
## sample estimates:
## prop 1 prop 2
## 0.7635 0.7610

## [1] "CF11 AUM2 CL vs secondary Ab PDL1"

##
## 2-sample test for equality of proportions with continuity correction
##
## data:  c(7397, 7610) out of c(10000, 10000)
## X-squared = 11.996, df = 1, p-value = 0.0002665
## alternative hypothesis: less
## 95 percent confidence interval:
##  -1.00000000 -0.01113512
## sample estimates:
## prop 1 prop 2
## 0.7397 0.7610

## [1] "D22 AUM2 super vs secondary Ab PDL1"

##
## 2-sample test for equality of proportions with continuity correction
##
## data:  c(7225, 7610) out of c(10000, 10000)
## X-squared = 38.489, df = 1, p-value = 2.753e-10
## alternative hypothesis: less
## 95 percent confidence interval:
##  -1.00000000 -0.02822883
## sample estimates:
## prop 1 prop 2
## 0.7225 0.7610

## [1] "D22 AUM2 CL vs secondary Ab PDL1"

##
##  2-sample test for equality of proportions with continuity correction
```

```
##
## data:  c(7401, 7610) out of c(10000, 10000)
## X-squared = 11.554, df = 1, p-value = 0.000338
## alternative hypothesis: less
## 95 percent confidence interval:
##  -1.0000000 -0.0107377
## sample estimates:
## prop 1 prop 2
## 0.7401 0.7610

## [1] "MC-KOS AUM2 super vs secondary Ab PDL1"

##
## 2-sample test for equality of proportions with continuity correction
##
## data:  c(8072, 7610) out of c(10000, 10000)
## X-squared = 62.769, df = 1, p-value = 1
## alternative hypothesis: less
## 95 percent confidence interval:
##  -1.00000000  0.05585584
## sample estimates:
## prop 1 prop 2
## 0.8072 0.7610

## [1] "MC-KOS AUM2 CL vs secondary Ab PDL1"

##
## 2-sample test for equality of proportions with continuity correction
##
## data:  c(7735, 7610) out of c(10000, 10000)
## X-squared = 4.3051, df = 1, p-value = 0.981
## alternative hypothesis: less
## 95 percent confidence interval:
##  -1.00000000  0.02242897
## sample estimates:
## prop 1 prop 2
## 0.7735 0.7610
```
